# Indirect acute effects of the COVID-19 pandemic on physical and mental health in the UK: a population-based study

**DOI:** 10.1016/S2589-7500(21)00017-0

**Published:** 2021-02-18

**Authors:** Kathryn E Mansfield, Rohini Mathur, John Tazare, Alasdair D Henderson, Amy R Mulick, Helena Carreira, Anthony A Matthews, Patrick Bidulka, Alicia Gayle, Harriet Forbes, Sarah Cook, Angel Y S Wong, Helen Strongman, Kevin Wing, Charlotte Warren-Gash, Sharon L Cadogan, Liam Smeeth, Joseph F Hayes, Jennifer K Quint, Martin McKee, Sinéad M Langan

**Affiliations:** a Department of Non-Communicable Disease Epidemiology, London School of Hygiene & Tropical Medicine, London, UK; b Department of Health Services Research and Policy, London School of Hygiene & Tropical Medicine, London, UK; c Unit of Epidemiology, Institute of Environmental Medicine, Karolinska Institutet, Stockholm, Sweden; d National Heart and Lung Institute, Imperial College London, London, UK; e Population Health Sciences, Bristol Medical School, University of Bristol, Bristol, UK; f Department of Community Medicine, University of Tromsø—The Arctic University of Norway, Tromsø, Norway; g Division of Psychiatry, University College London, London, UK; h Health Data Research UK, London, UK

## Abstract

**Background:**

There are concerns that the response to the COVID-19 pandemic in the UK might have worsened physical and mental health, and reduced use of health services. However, the scale of the problem is unquantified, impeding development of effective mitigations. We aimed to ascertain what has happened to general practice contacts for acute physical and mental health outcomes during the pandemic.

**Methods:**

Using de-identified electronic health records from the Clinical Research Practice Datalink (CPRD) Aurum (covering 13% of the UK population), between 2017 and 2020, we calculated weekly primary care contacts for selected acute physical and mental health conditions: anxiety, depression, self-harm (fatal and non-fatal), severe mental illness, eating disorder, obsessive-compulsive disorder, acute alcohol-related events, asthma exacerbation, chronic obstructive pulmonary disease exacerbation, acute cardiovascular events (cerebrovascular accident, heart failure, myocardial infarction, transient ischaemic attacks, unstable angina, and venous thromboembolism), and diabetic emergency. Primary care contacts included remote and face-to-face consultations, diagnoses from hospital discharge letters, and secondary care referrals, and conditions were identified through primary care records for diagnoses, symptoms, and prescribing. Our overall study population included individuals aged 11 years or older who had at least 1 year of registration with practices contributing to CPRD Aurum in the specified period, but denominator populations varied depending on the condition being analysed. We used an interrupted time-series analysis to formally quantify changes in conditions after the introduction of population-wide restrictions (defined as March 29, 2020) compared with the period before their introduction (defined as Jan 1, 2017 to March 7, 2020), with data excluded for an adjustment-to-restrictions period (March 8–28).

**Findings:**

The overall population included 9 863 903 individuals on Jan 1, 2017, and increased to 10 226 939 by Jan 1, 2020. Primary care contacts for almost all conditions dropped considerably after the introduction of population-wide restrictions. The largest reductions were observed for contacts for diabetic emergencies (odds ratio 0·35 [95% CI 0·25–0·50]), depression (0·53 [0·52–0·53]), and self-harm (0·56 [0·54–0·58]). In the interrupted time-series analysis, with the exception of acute alcohol-related events (0·98 [0·89–1·10]), there was evidence of a reduction in contacts for all conditions (anxiety 0·67 [0·66–0·67], eating disorders 0·62 [0·59–0·66], obsessive-compulsive disorder [0·69 [0·64–0·74]], self-harm 0·56 [0·54–0·58], severe mental illness 0·80 [0·78–0·83], stroke 0·59 [0·56–0·62], transient ischaemic attack 0·63 [0·58–0·67], heart failure 0·62 [0·60–0·64], myocardial infarction 0·72 [0·68–0·77], unstable angina 0·72 [0·60–0·87], venous thromboembolism 0·94 [0·90–0·99], and asthma exacerbation 0·88 [0·86–0·90]). By July, 2020, except for unstable angina and acute alcohol-related events, contacts for all conditions had not recovered to pre-lockdown levels.

**Interpretation:**

There were substantial reductions in primary care contacts for acute physical and mental conditions following the introduction of restrictions, with limited recovery by July, 2020. Further research is needed to ascertain whether these reductions reflect changes in disease frequency or missed opportunities for care. Maintaining health-care access should be a key priority in future public health planning, including further restrictions. The conditions we studied are sufficiently severe that any unmet need will have substantial ramifications for the people with the conditions as well as health-care provision.

**Funding:**

Wellcome Trust Senior Fellowship, Health Data Research UK.

## Introduction

By January, 2021, COVID-19 had been diagnosed in more than 100 million individuals, with over 2 million deaths reported worldwide.^1^ Much research and public health attention has, understandably, focused on preventing infection with SARS-CoV-2 and reducing mortality. However, there are concerning reports of decreased health service use.^2^, ^3^, ^4^, ^5^ Inevitably, there will be effects on non-COVID-19-related health-care provision, with health-care resources reallocated to the COVID-19 response and care delivery modified because of mitigation measures including physical distancing.^6^, ^7^, ^8^, ^9^, ^10^, ^11^ Additionally, individuals might have delayed seeking care during the pandemic (due to fear of infection or to avoid burdening health services). Psychological health will have been affected by pandemic-related fears, employment and financial concerns, and control measures (including physical distancing, closures of social spaces, and isolation),^12^, ^13^ and lockdown measures are likely to have reduced access to mental health care (face-to-face visits and talking therapies). Understanding the indirect effects of the pandemic and its control measures is essential for public health planning, particularly when and if the COVID-19 pandemic is under control (or if further restrictions are needed), and for informing control measures for future pandemics.

**Research in context**

**Evidence before this study**

A small study in 47 general practitioners' practices in a largely deprived, urban area of the UK (Salford) reported that primary care consultations for four broad diagnostic groups (circulatory disease, common mental health problems, type 2 diabetes, and malignant cancer) declined by 16–50% between March and May, 2020, compared with what was expected based on data from January, 2010, to March, 2020. We searched MEDLINE for other relevant evidence of the indirect effect of the COVID-19 pandemic on physical and mental health, from inception to Sept 25, 2020, for articles published in English, with titles including the search terms (“covid*” or “coronavirus” or “sars-cov-2”), and title or abstracts including the search terms (“indirect impact” or “missed diagnos*” or “missing diagnos*” or “delayed diagnos*” or ((“present*” or “consult*” or “engag*” or “access*”) AND (“reduction” or “decrease” or “decline”)). We found no further studies investigating the change in primary care contacts for specific physical and mental health conditions indirectly resulting from the COVID-19 pandemic or its control measures. There has been a reduction in hospital admissions and presentations to accident and emergency departments in the UK, particularly for myocardial infarctions and cerebrovascular accidents. However, there is no published evidence specifically investigating the changes in primary care contacts for severe acute physical and mental health conditions.

**Added value of this study**

To our knowledge this is the first study to explore changes in health-care contacts for acute physical and mental health conditions in a large population representative of the UK. We used electronic primary care health records of around 10 million individuals across the UK to investigate the indirect effects of the pandemic on primary care contacts for mental health, acute alcohol-related events, asthma and chronic obstructive pulmonary disease (COPD) exacerbations, and cardiovascular and diabetic emergencies up to July, 2020. For all conditions studied, we found primary care contacts dropped dramatically after the introduction of population-wide restriction measures in March, 2020. By July, 2020, with the exception of unstable angina and acute alcohol-related events, primary care contacts for all conditions studied had not recovered to pre-lockdown levels. In the general population, estimates of the absolute reduction in the number of primary care contacts cumulatively to July, 2020, compared with what we would expect from previous years, varied from fewer than ten contacts per million for some cardiovascular outcomes, to 6600 per million for anxiety and 12 800 per million for depression. In people with COPD, we estimated 43 900 per million fewer contacts for COPD exacerbations to July, 2020, than what we would expect from previous years.

**Implications of all the available evidence**

Although our results might represent some genuine reduction in disease frequency (eg, the restriction measures could have improved diabetic glycaemic control through more regular daily routines at home), it is more likely the reduced primary care contacts we saw represent a substantial burden of unmet need (particularly for mental health conditions) that could be reflected in subsequent increased mortality and morbidity. Health service providers should take steps to prepare for increased demand in the coming months and years, due to the short-term and long-term ramifications of reduced access to care for severe acute physical and mental health conditions. Maintaining access to primary care is key to future public health planning in relation to the pandemic.

Reports indicate that accident and emergency department attendance and hospital admissions for non-COVID-19-related acute concerns in the UK have declined since March, 2020.^2^, ^3^, ^4^ However, it is not yet clear what has happened in primary care across the UK where clinical work has changed rapidly to include more remote consultations,^14^, ^15^, ^16^, ^17^ although a regional report indicates reduced primary care consultations.^18^

To inform decisions on policy responses and resource allocation, we asked how primary care contacts (including face-to-face or remote consultations and recording of diagnoses from hospital discharge summaries) have changed for selected indirect acute physical and mental health effects of the COVID-19 pandemic. Although a wide range of diagnoses could be indirectly affected by the pandemic, we focused on specific acute conditions that could plausibly be affected, including mental health conditions, acute alcohol-related events, cardiovascular and diabetic emergencies, and asthma and chronic obstructive pulmonary disease (COPD) exacerbations. We specifically selected diabetic and cardiovascular emergencies (including myocardial infarction and unstable angina) as well as asthma and COPD exacerbations because affected individuals are likely to be considered vulnerable and thus advised to shield (ie, to avoid unnecessary contacts to avoid infection),^19^ creating a barrier to accessing health-care resources.

## Methods

### Study overview and data source

We analysed routinely collected primary care data from electronic health records from general practices that contributed to the Clinical Research Practice Datalink (CPRD) Aurum database (August, 2020 build) during the period from Jan 1, 2017 to July 18, 2020—ie, 3 years before the COVID-19 pandemic and 4 months after the introduction of population-wide restrictions (lockdown) in the UK on March 23, 2020 (appendix p 1).^20^ CPRD Aurum includes de-identified data from participating general practices covering 13% of the UK population, and is broadly representative of the English population with respect to age, sex, ethnicity, and geographical region.^20^ Individuals registered at consenting practices in England from 2017 and Northern Irish practices from 2019 are included in the database.

Code lists for defining all outcomes and stratifying variables and analytical code are available online.

The study was approved by the London School of Hygiene & Tropical Medicine Research Ethics Committee (reference 22143 /RR/18495) and by the CPRD Independent Scientific Advisory Committee (protocol number 20_089R2).

### Study population

Our overall study population included individuals aged 11 years or older who had at least 1 year of registration with practices contributing to CPRD Aurum in the specified period. Included populations (denominators) varied depending on the condition being investigated (table 1; appendix p 2). For example, for diabetic emergencies, the denominator population only included individuals aged 11 years or older with an existing diabetes diagnosis, whereas the denominator population for myocardial infarction was all individuals from the overall study population aged 31 years or older.

**Table 1:** Description of denominator populations and condition definitions

		**Condition-specific denominator population**	**Condition definition**
Diabetic emergency		All individuals (aged ≥11 years) with prevalent diagnoses of diabetes at the start of each week of follow-up; individuals contributed to the study population from whichever was latest of the start of follow-up in the overall population and the date of their first record indicating a diagnosis of diabetes	Any record of diabetes-related hyperglycaemia, hypoglycaemia, ketoacidosis, or diabetic coma. Multiple records occurring within 7 days of each other were considered to represent the same event
Mental health conditions			
	Anxiety	All individuals (aged ≥11 years) from the overall study population	Any record of symptoms or diagnoses of social phobia, agoraphobia, panic, generalised anxiety disorder, and mixed anxiety and depression; multiple records occurring within 7 days of each other were considered to represent the same event
	Depression	All individuals (aged ≥11 years) from the overall study population	Any record of major depressive disorder, dysthymia, mixed anxiety and depression, and adjustment disorders with depressed mood; we also included codes for depressive symptoms; multiple records occurring within 7 days of each other were considered to represent the same event
	Self-harm	All individuals (aged ≥11 years) from the overall study population	Records that indicated explicit or undetermined intention to self-harm, non-suicidal or suicidal self-harm (including overdoses with drugs commonly implicated in suicide, such as paracetamol); multiple records occurring within 7 days of each other were considered to represent the same event
	Serious mental illness	All individuals (aged ≥11 years) from the overall study population	Diagnoses of schizophrenia and other psychotic disorders, and bipolar disorders; multiple records occurring within 7 days of each other were considered to represent the same event
	Eating disorder	All individuals (aged ≥11 years) from the overall study population	Anorexia nervosa, bulimia nervosa, and other specified feeding and eating disorders; multiple records occurring within 7 days of each other were considered to represent the same event
	Obsessive-compulsive disorder	All individuals (aged ≥11 years) from the overall study population	Codes for body dysmorphic disorders, hypochondriasis, hoarding disorder, and body focused repetitive behaviour disorders; multiple records occurring within 7 days of each other were considered to represent the same event
Acute respiratory events			
	Asthma exacerbation	All individuals (aged ≥11 years) with a current asthma diagnosis (ie, asthma code in the past 2 years if aged <18 years or the past 3 years if aged ≥18 years); individuals joined the study population from the start of follow-up in the overall population if there was a current asthma diagnosis (within past 2–3 years) at that time, or from the date of their first record indicating an asthma diagnosis within the overall follow-up period; participants remained in the study until there was no current asthma diagnosis or until the end of overall follow-up; they could re-enter the study if there was a later diagnostic code for asthma before the end of overall follow-up; following an existing definition, individuals aged ≥40 years with asthma were considered likely to have COPD (and therefore not included in the asthma denominator population) if they had a subsequent COPD diagnosis recorded within the 2 years following the current asthma record^21^	Records for morbidity codes for asthma exacerbations and status asthmaticus, and a primary care prescription for an oral corticoseroid;^22^ multiple records occurring within 14 days of each other were considered to represent the same event
	COPD exacerbation	Adults (aged ≥41 years) with an established diagnosis of COPD and evidence of a smoking history;^23^ individuals joined the study population from whichever was latest of the start of follow-up in the overall population and the date of their first record indicating diagnosis of COPD	Morbidity codes (in individuals with existing COPD) for COPD exacerbations, lower respiratory tract infections, breathlessness or sputum production, and a new prescription for an oral corticosteroid or antibiotic;^24^ multiple records occurring within 14 days of each other were considered to represent the same event
Acute cardiovascular events			
	Myocardial infarction	All adults (aged ≥31 years)	Any record for myocardial infarction allowing for a 1-year window between successive records; multiple records occurring within 1 year of each other were considered to represent the same event
	Unstable angina	All adults (aged ≥31 years)	Any record for unstable angina, allowing for a 6-month window between successive records; multiple records occurring within 6 months of each other were considered to represent the same event
	Transient ischaemic attacks	All adults (aged ≥31 years)	Any record for transient ischaemic attack, allowing for a 6-month window between successive records; multiple records occurring within 6 months of each other were considered to represent the same event
	Stroke	All adults (aged ≥31 years)	Any record for stroke, allowing for a 1-year window between successive records; multiple records occurring within 1 year of each other were considered to represent the same event
	Cardiac failure	All adults (aged ≥31 years)	Given the complexity with capturing acute events for a chronic condition, we only counted an individual's first ever diagnosis with cardiac failure
	Venous thromboembolism (pulmonary embolism and deep venous thrombosis)	All adults (aged ≥31 years)	Any record for venous thromboembolism, allowing for a 1-year window between successive records; multiple records occurring within 1 year of each other were considered to represent the same event
Acute alcohol-related event		All adults (aged ≥18 years)	Any record for acute physical or psychological alcohol-related event, including acute alcoholic pancreatitis; multiple records occurring within 14 days of each other were considered to represent the same event

COPD=chronic obstructive pulmonary disease.

We followed all individuals from whichever was later of the following: the study start date (Jan 1, 2017), 1 year from registration with a general practitioner (GP), or (where applicable) from meeting our definitions for having diabetes or respiratory disease (table 1). Follow-up ended for all study populations at the earliest of the following: end of registration with GP, death, end of the practice contributing to CPRD, or end of the study period (July 18, 2020, chosen as most recent data available).

### Exposures, outcomes, and stratifying variables

Our exposure was the introduction of lockdown in the UK on March 23, 2020. As outcomes, we considered the number of weekly primary care contacts for the following conditions separately: mental health (depression, anxiety, fatal and non-fatal self-harm, severe mental illness, eating disorders, and obsessive-compulsive disorder), acute alcohol-related event, diabetic emergency (eg, ketoacidosis), asthma exacerbation, COPD exacerbation, and acute cardiovascular events (unstable angina, myocardial infarction, transient ischaemic attack, stroke, cardiac failure, and venous thromboembolisms). We used the term “contact” broadly to represent remote and face-to-face consultations, diagnoses from hospital discharge letters, and secondary care referrals. We identified conditions through primary care records for diagnoses, symptoms, and prescribing (table 1). All outcomes, except asthma and COPD exacerbations, were captured on the basis of the presence or absence of specific morbidity codes. Asthma and COPD exacerbations were based on validated algorithms requiring a combination of specific morbidity codes and prescriptions for corticosteroids or (for COPD) antibiotics.^22^, ^24^ For some conditions, we defined an exclusion period during which we regarded further coding for the same outcome as representing the same acute event (eg, for diabetic emergencies we regarded multiple records within 7 days of each other as representing the same event). We used different condition-specific periods to define outcome events to account for differences in natural history of study outcomes (table 1).

We stratified on the following prespecified variables: age (in 10-year bands), sex, geographical region, and ethnicity (appendix p 3).

### Statistical analysis

We described all denominator study populations in the first week of January for each year from 2017 to 2020. We plotted the percentage of our study populations with contacts for particular conditions in the given weeks in 2020 and the historical averages for that week from 2017 to 2019. We repeated analyses stratified by age, sex, region, and ethnicity.

To quantify changes in consultation behaviour following the introduction of restrictions, we used an interrupted time-series analysis, separating our time series into two periods: a pre-lockdown period (Jan 1, 2017, to March 7, 2020) for all outcomes except self-harm (which excluded data from 2017 and 2018; appendix p 12); and a with-restrictions period (March 29 to July 18, 2020).

Although restrictions were announced on March 23,^25^ public activity levels (measured by mobile phone applications and public transport journeys) had declined before the announcement.^26^, ^27^, ^28^ To account for anticipatory behaviour, we conservatively defined the start of restrictions as March 8, 2020 and removed data for 3 weeks in March up to and including the week restrictions were announced (March 8–28, 2020, inclusive) from this analysis.

For our interrupted time-series analysis, we used binomial generalised linear models with number of weekly contacts weighted by dynamic population sizes (updated weekly).^29^ We included a linear effect of time to capture long-term behaviour trends, a binary pre-lockdown or with-restrictions variable to measure the direct step change in behaviour, and an interaction between the two to allow for a recovery slope change in behaviour. We accounted for seasonal effects by including calendar month as a categorical variable, and autocorrelation by including first-order lagged residuals. Standard errors were scaled to account for overdispersion.^30^

To estimate the reduction in contacts as restrictions were introduced (the step change), we calculated odds ratios (ORs) for the relative difference in contacts at the start of the with-restrictions period compared with the end of the pre-lockdown period. To estimate the recovery of contacts over time (the slope), we used the coefficients from the interrupted time-series model to estimate the weekly log odds of contact during the with-restrictions period (appendix p 16).

To estimate absolute effects of restrictions on the number of contacts, we repeated our analysis using Poisson regression to generate linear predictions of the estimated log contact count and the estimated log count if the restrictions term was set to zero (ie, there had been no restrictions). To quantify absolute changes in behaviour over time, we compared the point estimate of the estimated number of contacts with and without restrictions during two 1-week periods: 1 month (April 26) and 3 months (June 28) from the start of the with-restrictions period.

We used Stata version 16 and R version 4.0.2 for our analyses.

Because our definitions for pre-lockdown and with-restrictions periods might have influenced our estimates, we did sensitivity analyses in which we repeated the interrupted time-series analysis with the same pre-lockdown period (until March 7) but with variable data-exclusion periods (5 weeks [March 8 to April 11]and 7 weeks [March 8 to April 25], versus 3 weeks in the main analysis). We also repeated analyses with the pre-lockdown period ending on March 21 (the week restrictions were announced)^25^ and with data excluded for 0 weeks (no adjustment-to-restrictions period, with-restrictions period March 22 to July 18, 2020), 3 weeks (March 22 to April 11), 5 weeks (March 22 to April 25), and 7 weeks (March 22 to May 9) as sensitivity analyses. Additionally, given the small number of diabetic emergency contacts, we varied our definition using less specific codes in a post-hoc sensitivity analysis (appendix p 26).

### Role of the funding source

The funders of the study had no role in study design, data collection, data analysis, data interpretation, or writing of the report.

## Results

The overall denominator population included 9 863 903 individuals on Jan 1, 2017, and numbers remained relatively stable throughout the study (table 2). The characteristics of condition-specific study populations are shown in the appendix (pp 4–8).

**Table 2:** General denominator population defined in the first week of each year from 2017 to 2020

	**2017 (n=9 863 903)**	**2018 (n=10 124 026)**	**2019 (n=10 286 472)**	**2020 (n=10 226 939)**
**Age, years**				
11–20	1 233 387 (13%)	1 283 296 (13%)	1 319 983 (13%)	1 325 412 (13%)
21–30	1 455 550 (15%)	1 499 066 (15%)	1 517 439 (15%)	1 505 172 (15%)
31–40	1 559 933 (16%)	1 622 838 (16%)	1 662 883 (16%)	1 661 724 (16%)
41–50	1 577 507 (16%)	1 579 296 (16%)	1 573 889 (15%)	1 550 104 (15%)
51–60	1 520 720 (15%)	1 564 290 (15%)	1 590 738 (15%)	1 580 348 (15%)
61–70	1 165 390 (12%)	1 166 078 (12%)	1 176 134 (11%)	1 164 688 (11%)
71–80	833 570 (8%)	881 099 (9%)	907 289 (9%)	904 486 (9%)
81–90	426 769 (4%)	436 646 (4%)	445 112 (4%)	442 098 (4%)
91–100	91 077 (1%)	91 417 (1%)	93 005 (1%)	92 907 (1%)
**Ethnicity**				
White	4 814 510 (49%)	4 965 265 (49%)	5 076 482 (49%)	4 996 494 (49%)
South Asian	425 917 (4%)	452 344 (4%)	463 579 (5%)	479 777 (5%)
Black	261 552 (3%)	273 841 (3%)	276 359 (3%)	282 515 (3%)
Other	147 583 (1%)	162 963 (2%)	177 156 (2%)	188 423 (2%)
Mixed	94 174 (1%)	102 384 (1%)	109 025 (1%)	114 211 (1%)
Missing	4 120 167 (42%)	4 167 229 (41%)	4 183 871 (41%)	4 165 519 (41%)
**Sex**				
Female	4 921 693 (50%)	5 046 616 (50%)	5 126 260 (50%)	5 092 370 (50%)
Male	4 942 210 (50%)	5 077 410 (50%)	5 160 212 (50%)	5 134 569 (50%)
**Region**				
North East	343 510 (3%)	348 039 (3%)	353 452 (3%)	342 460 (3%)
North West	1 690 063 (17%)	1 723 286 (17%)	1 753 263 (17%)	1 767 506 (17%)
Yorkshire and the Humber	371 809 (4%)	381 620 (4%)	390 222 (4%)	359 872 (4%)
East Midlands	259 468 (3%)	268 087 (3%)	278 011 (3%)	233 006 (2%)
West Midlands	1 571 832 (16%)	1 603 107 (16%)	1 602 242 (16%)	1 625 072 (16%)
East of England	464 376 (5%)	472 509 (5%)	472 546 (5%)	433 438 (4%)
South West	1 185 045 (12%)	1 216 271 (12%)	1 217 968 (12%)	1 204 833 (12%)
South Central	1 242 192 (13%)	1 271 663 (13%)	1 289 755 (13%)	1 303 108 (13%)
London	1 842 724 (19%)	1 929 942 (19%)	1 995 412 (19%)	2 027 364 (20%)
South East Coast	827 239 (8%)	842 833 (8%)	867 299 (8%)	862 929 (8%)
Northern Ireland	47 713 (<1%)	48 759 (<1%)	49 767 (<1%)	50 825 (<1%)

Data are n (%).

**Figure 1:**
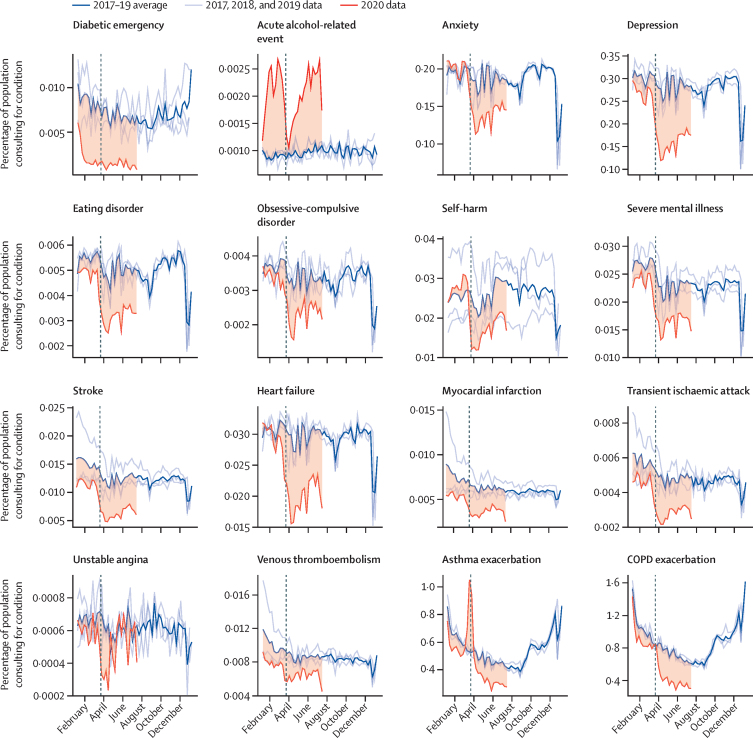
Proportions of each study population with contacts for each condition in 2017–19 and 2020 Percentage of eligible population with contacts for each health condition studied in 2020 compared with the historical (2017–19) average for that week. Shaded regions show the difference between the 2020 data and the historical average. Vertical dashed lines indicate the introduction of lockdown restrictions in the UK on March 23, 2020. Tick marks on the x-axis represent the first day of the specified month. COPD=chronic obstructive pulmonary disease.

**Figure 2:**
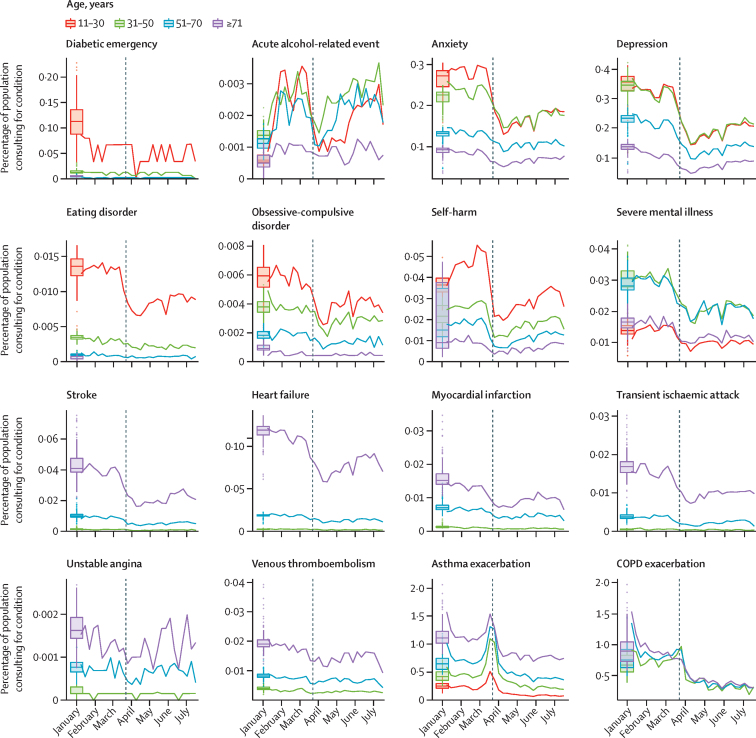
Percentage of each denominator population with general practitioner contacts for the study conditions throughout 2020, by age group Coloured lines represent weekly percentages of the eligible population with primary care contacts for the condition of interest in 2020; eligible populations differed by condition (table 1). Boxplots represent the historical average (median and IQR) percentage of the study population with general practitioner contacts for the condition of interest. Vertical dashed lines indicate the introduction of lockdown restrictions in the UK on March 23, 2020. Tick marks on the x-axis represent the first day of the specified month. Note that cell counts with fewer than five contacts in 1 week in 2020 have been suppressed. COPD=chronic obstructive pulmonary disease.

Figure 1 shows the percentage of a given study population with primary care contacts for each condition in 2020 and a 3-year historical average for the corresponding week. Across the majority of conditions, we observed rapid and sustained decreases in GP contacts between March and July, 2020, compared with pre-lockdown periods. Despite gradual increases in contacts as a percentage of denominator population following restrictions, levels remained below the 3-year average for all conditions except acute alcohol-related events (which were higher than the historical average in 2020) and unstable angina. During March, 2020, we observed pronounced increases in contacts related to asthma exacerbations. Patterns were broadly consistent when stratified by age (figure 2), sex, region, and ethnicity (appendix pp 9–11).

**Figure 3:**
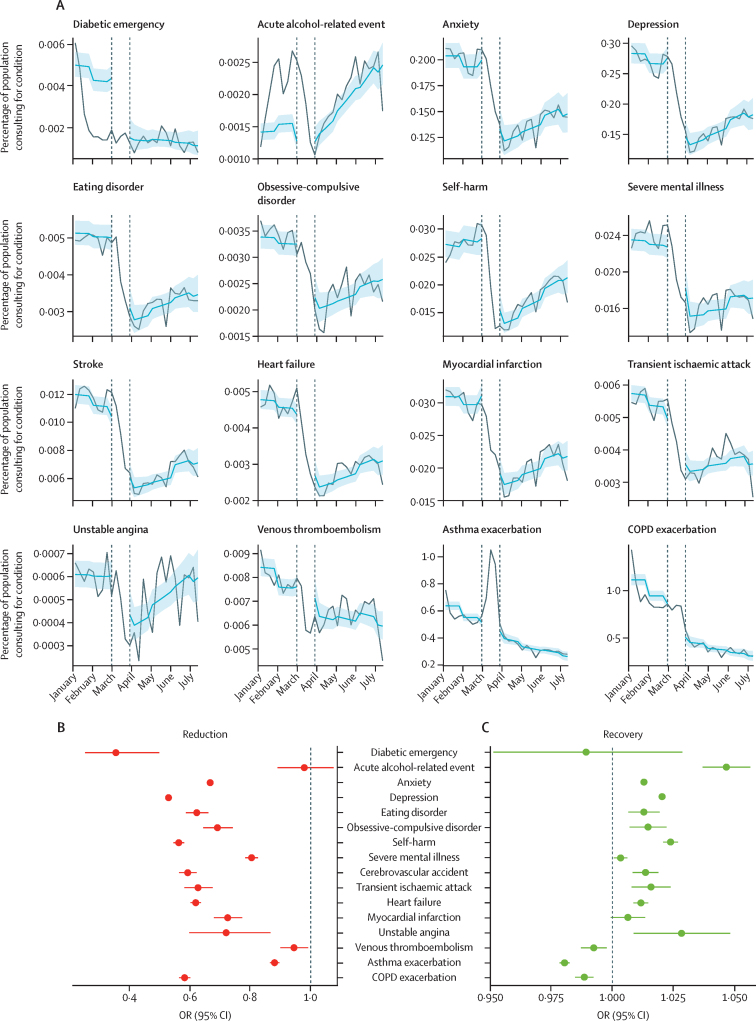
Interrupted time-series analysis of changes in general practitioner contacts before and after the introduction of UK-wide restrictions (A) Lines indicate the observed percentage of the denominator population with primary care contacts for each health condition in 2020. Shaded regions indicate the predicted percentage of contacts from the full interrupted time-series model (including data from 2017 onwards). Vertical lines show the adjustment-to-restrictions period from which data were excluded from the analysis (March 8–28, 2020). Tick marks on the x-axis represent the first day of the specified month. (B) 95% CIs of ORs for the estimated relative reduction in contacts as a percentage of the denominator population for each health condition immediately after the adjustment-to-restrictions period (March 29, 2020) compared with the pre-lockdown period (values closer to 0 represent a greater reduction in the estimated percentage of people with general practitioner contacts). (C) 95% CIs of ORs for the estimated effect of time (in weekly increments) since the introduction of restrictions (March 29, 2020)on contacts as a percentage of the denominator population for each condition (values >1 indicate an increasing percentage of population with contacts over time). Results for 2020 only are shown here (see appendix p 24 for full model fit to data from 2017, and appendix pp 17–18 for full relative reduction and recovery ORs and 95% CIs). COPD=chronic obstructive pulmonary disease. OR=odds ratio.

There was evidence that contacts for all studied conditions, except acute alcohol-related events, were lower after restrictions were announced compared with pre-restriction levels (figure 3A). The largest relative reductions in contact behaviour following restriction introduction were observed for diabetic emergencies (OR 0·35 [95% CI 0·25–0·50]), depression (0·53 [0·52–0·53]), and self-harm (0·56 [0·54–0·58]). With the exception of acute alcohol-related events (0·98 [0·89–1·10]), there was evidence of a reduction in contact behaviour for all conditions studied: anxiety 0·67 (0·66–0·67), eating disorders 0·62 (0·59–0·66), obsessive-compulsive disorder (0·69 [0·64–0·74]), self-harm 0·56 (0·54–0·58), severe mental illness 0·80 (0·78–0·83), stroke 0·59 (0·56–0·62), transient ischaemic attack 0·63 (0·58–0·67), heart failure 0·62 (0·60–0·64), myocardial infarction 0·72 (0·68–0·77), unstable angina 0·72 (0·60–0·87), venous thromboembolism 0·94 (0·90–0·99), and asthma exacerbation 0·88 (0·86–0·90; figure 3B; appendix p 17).

From March 29, 2020, we saw evidence of increasing contacts for most conditions over time. Acute alcohol-related events and unstable angina contacts appeared to recover faster (3–5% increase in odds of contact per week; figure 3C; appendix p 18) than, for example, mental health contacts, for which odds of contact increased by 1–2% per week despite a 20–47% drop following restrictions (figure 3B; appendix p 17). Sensitivity analyses using varying exclusion periods between pre-lockdown and with-restrictions periods provided broadly consistent results over a range of scenarios (appendix pp 17–25).

Table 3 shows the potential impact of reduced contacts on relevant populations. For some rare conditions, such as unstable angina and acute alcohol-related events, the absolute change in contacts was relatively small; however, other more common conditions had a larger absolute change in contacts. For example, compared with expected numbers of COPD exacerbation contacts per million people with COPD, we estimated that there were cumulatively 43 900 fewer contacts between March 29 and July 4; there were 3640 fewer contacts from April 26 to June 2 and 3230 fewer from June 28 to July 4, indicating a slow return to pre-lockdown contact levels but not complete recovery. Cumulatively between March 29 and July 4, we also estimated 14 100 fewer asthma exacerbation contacts for every million people with asthma, 12 800 fewer depression contacts per million people in the denominator population, and 6600 fewer anxiety contacts per million people in the denominator population.

**Table 3:** Estimated reduction in number of primary care contacts

	**Estimated number of contacts per week per 1 million people in denominator population (95% CI)**		Difference in estimated number of contacts per 1 million people*	**Cumulative sum of difference in primary care contacts since March 29, 2020**†
	Without COVID-19 and restrictions	With COVID-19 and restrictions		
**Diabetic emergency**				
April 26–May 2	39 (34–44)	14 (10–20)	<100	<100
June 28–July 4	38 (33–43)	12 (8–19)	<100	330
**Acute alcohol-related event**				
April 26–May 2	13 (11–14)	16 (15–18)	>–10	>–100
June 28–July 4	14 (13–16)	24 (21–26)	>–10	>–100
**Anxiety**				
April 26–May 2	1816 (1695–1945)	1266 (1148–1396)	550	2300
June 28–July 4	1943 (1818–2076)	1532 (1383–1696)	411	6600
**Depression**				
April 26–May 2	2451 (2285–2629)	1391 (1241–1558)	1060	4440
June 28–July 4	2657 (2484–2843)	1857 (1657–2080)	801	12 800
**Eating disorder**				
April 26–May 2	44 (41–47)	29 (26–33)	<100	<100
June 28–July 4	47 (44–51)	35 (31–39)	<100	184
**Obsessive-compulsive disorder**				
April 26–May 2	29 (27–31)	22 (19–24)	<10	<100
June 28–July 4	30 (28–33)	25 (23–29)	<10	<100
**Self-harm**				
April 26–May 2	217 (190–247)	145 (130–162)	<100	307
June 28–July 4	254 (226–285)	205 (184–228)	<100	870
**Severe mental illness**				
April 26–May 2	184 (173–196)	155 (142–169)	<100	119
June 28–July 4	203 (192–215)	172 (157–189)	<100	391
**Stroke**				
April 26–May 2	88 (83–94)	56 (50–62)	<100	135
June 28–July 4	100 (93–106)	73 (65–81)	<100	400
**Transient ischaemic attack**				
April 26–May 2	37 (35–40)	26 (24–29)	<100	<100
June 28–July 4	40 (38–43)	31 (28–35)	<10	136
**Heart failure**				
April 26–May 2	279 (264–295)	181 (167–196)	<100	408
June 28–July 4	308 (292–324)	223 (205–242)	<100	1240
**Myocardial infarction**				
April 26–May 2	45 (42–47)	35 (33–38)	<10	<100
June 28–July 4	47 (44–49)	37 (34–41)	<10	123
**Unstable angina**				
April 26–May 2	5 (5–6)	4 (4–5)	<10	<10
June 28–July 4	6 (5–6)	6 (5–7)	<10	<10
**Venous thromboembolism**				
April 26–May 2	67 (63–70)	64 (59–68)	<10	<10
June 28–July 4	72 (69–76)	63 (58–68)	<10	<100
**Asthma exacerbation**				
April 26–May 2	4636 (4361–4928)	3617 (3320–3941)	1020	3780
June 28–July 4	4254 (3995–4529)	2941 (2643–3273)	1310	14 100
**COPD exacerbation**				
April 26–May 2	7863 (7365–8395)	4222 (3768–4730)	3640	14 400
June 28–July 4	6594 (6147–7073)	3367 (2919–3884)	3230	43 900

Data represent the estimated number of primary care contacts for acute physical and mental health conditions in a hypothetical non-COVID-19 year compared with the number of contacts estimated from our model for 2020 for two week-long periods: April 26–May 2 and June 28–July 4. Estimates of the number of contacts are in a hypothetical population of 1 million people, but the reference populations are condition specific (table 1). COPD=chronic obstructive pulmonary disease.

* Difference in estimated number of contacts per million people in the specified week if pre-restriction trends in contacts had continued through the period with restrictions.

† Rounded to 3 significant figures to avoid overly precise estimates; we did not intend to estimate the exact number of missed consultations but obtained an estimate of the absolute indirect effect of COVID-19 on different conditions; if the expected difference was <100 or <10 then estimates have been censored for the same reason.

## Discussion

Primary care contacts for key physical and mental health conditions dropped considerably after the introduction of population-wide restriction measures in March, 2020. By July, 2020, with the exception of unstable angina and acute alcohol-related contacts, primary care contacts for all conditions studied remained below pre-lockdown levels. We estimated that by July, 2020, per million people in the general population, there were very small (<10) drops in the cumulative number of contacts for myocardial infarction, unstable angina, and venous thromboembolism. However, we estimated large drops for anxiety, depression, and COPD contacts.

Our study is the first to explore the effect of lockdown measures on primary care contacts for specific acute physical and mental health conditions across the UK. A study of 47 primary care practices in Salford, a largely deprived urban area in northwest England that was badly affected by the pandemic, suggested that primary care consultations across four broad categories (common mental health problems, cardiovascular and cerebrovascular disease, type 2 diabetes, and cancer) had reduced by up to 50% by the end of May, 2020.^18^ In contrast to the Salford study, our sample was nationally representative and focused on contacts for specific disease categories that we would expect to present to health-care providers. Our large sample size allowed us to investigate detailed diagnoses (for example, different types of cardiovascular disease and mental health conditions).

In September, 2020, GPs conducted more face-to-face appointments than any week since March, and more consultations overall than before the pandemic (40% were telephone appointments).^30^, ^31^ A study of 51 GP practices already offering remote consultations before the pandemic indicated a dip in overall consultations at the time of lockdown but, unlike our results for specific acute conditions, their post-lockdown overall consultation decrease was less extreme than that during the Christmas period of 2019.^32^ In England, there was a 30% decrease in GP consultations from the beginning to the end of March, 2020,^33^ with an increase in calls to NHS 111, the non-urgent telephone helpline. However, over 50% (1 573 835 of 2 962 751) of these calls went unanswered.^34^

The reduced diabetic emergency contacts we observed are consistent with the 49% reduction in new type 2 diabetes contacts (new prescriptions for metformin) in Salford. Although the Salford study highlighted missed new diagnoses, our study identifies missed contacts for acute deteriorations. Given that 90% of diabetes management is in primary care, the large relative reduction in the proportion of people with diabetes with diabetic emergency contacts is concerning.^35^

Recent evidence indicates a two-way interaction between diabetes and COVID-19, with a potentially causal association between COVID-19 infection and dysglycaemia, such that each condition exacerbates the other.^36^, ^37^ Furthermore, there is evidence that other emergency situations impair control of diabetes.^38^, ^39^, ^40^ Consequently, we would expect an increase, rather than decrease, in diabetic emergency contacts.

The reduction in cardiovascular disease contacts is consistent with reports from other UK studies.^18^, ^41^ Taken alongside findings of similar reductions in emergency department presentations and hospital admissions for cardiovascular outcomes in the UK, our findings highlight an area of major concern,^3^, ^42^ particularly as evidence from France indicates increased out-of-hospital cardiac arrest.^43^ Severe COVID-19 affects the cardiovascular system;^44^ therefore, increased primary and secondary care presentations for cardiovascular disease are expected.^45^ Indeed, it is possible that the more rapid recovery in unstable angina contacts (compared with other conditions included in our study) might reflect COVID-19-related cardiovascular disease. However, the number of unstable angina events recorded were small, so we are unable to draw any meaningful conclusions from these results.^46^

Reports from Germany, consistent with our findings, indicate reduced community and hospital presentations with acute COPD exacerbation.^47^ COPD is associated with more severe COVID-19,^48^ and individuals with COPD in the UK were recommended to avoid contact with others until September, 2020.^19^, ^49^

Decreased emergency department visits for childhood asthma have been reported in the USA, consistent with our observations.^50^ There is no compelling evidence that individuals with asthma are at greater risk of severe COVID-19 outcomes, although there was uncertainty at the onset of the pandemic.^51^, ^52^, ^53^ Viruses commonly trigger asthma exacerbations, so we might have expected to see more asthma contacts. Anecdotally, GPs reported increased prescription of asthma therapies around the lockdown period,^54^ which could explain initial increased asthma contacts. Similar increases in COPD exacerbation contacts were not seen around the introduction of restrictions, despite our definition including prescriptions for oral corticosteroids. One explanation might be that, as COPD is a progressive respiratory condition, individuals with COPD might have repeat prescriptions, reducing the need (compared with people with asthma) to stockpile drugs in a crisis.

Surveys have reported increased anxiety, depression, and self-harm during the pandemic,^12^, ^13^, ^55^, ^56^, ^57^ and exacerbations of existing obsessive-compulsive disorder, severe mental illness, and eating disorders have also been reported.^58^, ^59^, ^60^ However, we saw a sustained reduction in primary care contacts for anxiety, depression, and other mental health conditions consistent with other reports;^18^ this finding is concerning because the majority of common mental disorders are managed in primary care. Similarly, the observed reduction in health-care contacts for people with severe mental illness is concerning because these individuals are likely to be at greater risk of poor outcomes from COVID-19 because of the high prevalence of risk factors for adverse outcomes in this group (eg, cardiovascular disease and deprivation).^51^, ^61^, ^62^

Findings from surveys on alcohol consumption in lockdown have been mixed, with some reporting increased alcohol consumption in up to a third of people surveyed, while others had differing findings.^63^ We saw primary care contacts for acute alcohol-related events increase before and after restrictions, which is troubling given the reduction in contacts for other conditions studied; however, we urge caution in drawing robust conclusions as numbers were small.

This study involved a rapid assessment of changes in primary care contacts following the introduction of UK population-wide restrictions up to July, 2020, in a large sample representative of the UK population. Historical data allowed us to compare observed patterns in 2020 with trends in the previous 3 years. We estimated relative and absolute changes in contact patterns, with a focus on easy to interpret measures.

Our study describes and quantifies the reduction in primary care contacts across a wide range of health conditions likely to be affected by COVID-19 to generate hypotheses. However, further research is needed to understand the specific drivers behind these changes (eg, individuals could have limited their in-person contact through fear of SARS-CoV-2 infection, or might have had difficulty accessing primary care services because of unavailability of appointments or lack of available technology or technological literacy for virtual consultations). It is important that we understand what happened to individuals who did not consult their GP—specifically, whether they were treated in secondary care or self-managed, and to what extent our findings can be explained by genuine changes in disease frequency.

Without hospital and mortality data, we are unable to investigate whether, for example, any reduction in GP contacts resulted in corresponding increases in hospital attendances or deaths. We focused on studying any record of our conditions of interest, so our results reflect all primary care contacts, including diagnoses recorded by general practice staff from hospital discharge letters. Consequently, a potential explanation for our findings is that individuals with some of the emergency conditions studied might have presented directly to hospital for their emergency non-COVID-19 condition, with delayed recording of hospital discharge diagnoses in primary care health records as a result of changes in administrative practices in response to the pandemic restrictions. Similarly, we were unable to account for individuals with chronic conditions being admitted directly to hospital with SARS-CoV-2 infection. However, hospital COVID-19 admissions are unlikely to have resulted in the magnitude of the abrupt change in primary care contacts that we saw: hospital admissions for COVID-19 were increasing in March, 2020, but government data suggest that on March 27 there were 7043 individuals in hospital with a confirmed COVID-19 diagnosis,^64^ which would not account for the sudden and large decline in primary care contacts that we saw across most conditions studied.

Another potential explanation for our findings could be related to changes in how primary care contacts were documented following a rapid shift to remote consultations. However, we feel that the conditions we studied are sufficiently severe that it is unlikely that diagnoses would not have been recorded. To avoid problems arising from the timing of behavioural change associated with restrictions, our interrupted time-series analysis excluded a predefined intervention period when individuals' behaviours were changing dynamically. We took a conservative approach and defined our intervention period between March 8 and March 28, 2020, assuming that some people would have modified their behaviour before the introduction of restrictions. Sensitivity analyses varying the start date showed consistent findings with those of the main analysis.

Detailed exploration of whether consultation behaviour varied in people considered clinically vulnerable and advised to shield^18^ is beyond the scope of this Article, and any changes in health-seeking behaviour would not have reduced the need for care.

Given evidence suggesting reduced emergency department attendances and hospital admissions for our conditions of interest,^2^, ^3^, ^4^, ^5^ although one explanation could be genuine changes in disease frequency (which is unlikely, given consistent results across disease categories), it is more likely that our findings reflect missed opportunities for care. There are plausible mechanisms that might explain real reductions in frequency for some of our outcomes, such as better glycaemic control in diabetes because of more regular routines when staying home; less respiratory disease because of lower exposure to air pollution during lockdown,^65^ and reduced community-acquired respiratory infections because of shielding guidelines;^19^ and reduced alcohol consumption due to pub closures and reduced social contact. Conversely, there are plausible mechanisms that could explain genuine increased frequency of these conditions (eg, distress related to the pandemic affecting mental health and alcohol consumption, reduced exercise affecting cardiovascular health, changes in diet influencing glycaemic control). Additionally, for some of our outcomes, such as mental health conditions, some evidence indicates increased frequency.^12^, ^13^, ^55^, ^56^, ^58^, ^59^, ^60^ Increases in non-COVID-19-related excess mortality also make it more likely that our observed reduction in primary care contacts was due to behavioural changes rather than reduced disease frequency.^13^, ^66^, ^67^, ^68^, ^69^ Furthermore, emerging evidence of the systemic complications of SARS-CoV-2 infection (particularly cardiovascular disease and diabetes)^36^, ^70^, ^71^ indicates that we might have expected more need for care for these conditions as a direct result of the pandemic.

Our results are likely to represent a large burden of unmet need, particularly in relation to COPD and mental health conditions. Health-care providers should prepare for increases in morbidity and mortality in the coming months and years. Further research should address whether reduced clinical contact has resulted in excess mortality, and whether we need to increase service provision for individuals with increased health-care needs resulting from delaying seeking access to care. Although numbers of unstable angina events were small, we note a more rapid return to pre-pandemic consultation rates compared with that of other study outcomes; this observation needs investigation as it could be a direct consequence of the pandemic. Future research should also investigate potential behavioural drivers of the changes in primary care contacts we observed (eg, reluctance to initiate health-care contact, difficulty in making primary care appointments, or concerns about using information technology for remote consultations), as well as the effect of multiple periods of lockdown restrictions being imposed and lifted, and should include similar international studies to investigate the global implications of the pandemic on non-COVID-19 illness. Finally, our findings highlight a need to ensure equitable access to primary care in future pandemic planning, particularly with the added burden on primary care of vaccine delivery. Countries such as Singapore, which had experienced severe acute respiratory syndrome, implemented control measures in primary care rapidly.^72^ The current pandemic has generated a wealth of experience with alternative ways to access care remotely.^73^ These lessons must be systematised and implemented.

In summary, this study showed substantial reductions in primary care contacts for various acute physical and mental health conditions. Our findings are likely to represent a considerable burden of unmet need, which might lead to substantial increases in subsequent mortality and morbidity.

## Data sharing

No additional unpublished data are available as this study used existing data from the UK CPRD electronic health record database, which is only accessible to researchers with protocols approved by the CPRD's independent scientific advisory committee. All data management and analysis computer code is available via GitHub (see Methods). All code is shared without investigator support. Our study protocol and analysis plan are available in the appendix (pp 28–39). All aggregated data will be freely available to explore by stratifiers through an R Shiny app online.
