# Supplementary material for: Indirect acute effects of the COVID-19 pandemic on physical and mental health in the UK: a population-based study
Source: Lancet Digit Health. 2021 Feb 18;3(4):e217–30. doi: 10.1016/S2589-7500(21)00017-0 (PMC7985613; doi:10.1016/S2589-7500(21)00017-0)
Supplement: Supplementary appendix [file mmc1.pdf]

THE LANCET
Digital Health

Supplementary appendix

This appendix formed part of the original submission and has been peer reviewed. We post it as supplied by the authors.

Supplement to: Mansfield KE, Mathur R, Tazare J, et al. Indirect acute effects of the COVID-19 pandemic on physical and mental health in the UK: a population-based study. *Lancet Digit Health* 2021; published online Feb 18. https://doi.org/10.1016/S2589-7500(21)00017-0.

SUPPLEMENTARY MATERIAL

Figure S1. A simplified timeline illustrating the introduction and relaxation of key infection-control restriction measures in England in response to the COVID-19 pandemic, between January 2020 and July 2020.[1,2]

Blue region, pre-restrictions; Pink region, with restrictions; Region with no shading, the period 8th March to 28th March 2020 excluded from our interrupted-time-series analyses to account for rapid behavioural changes in response to the pandemic and measures introduced to limit its spread.

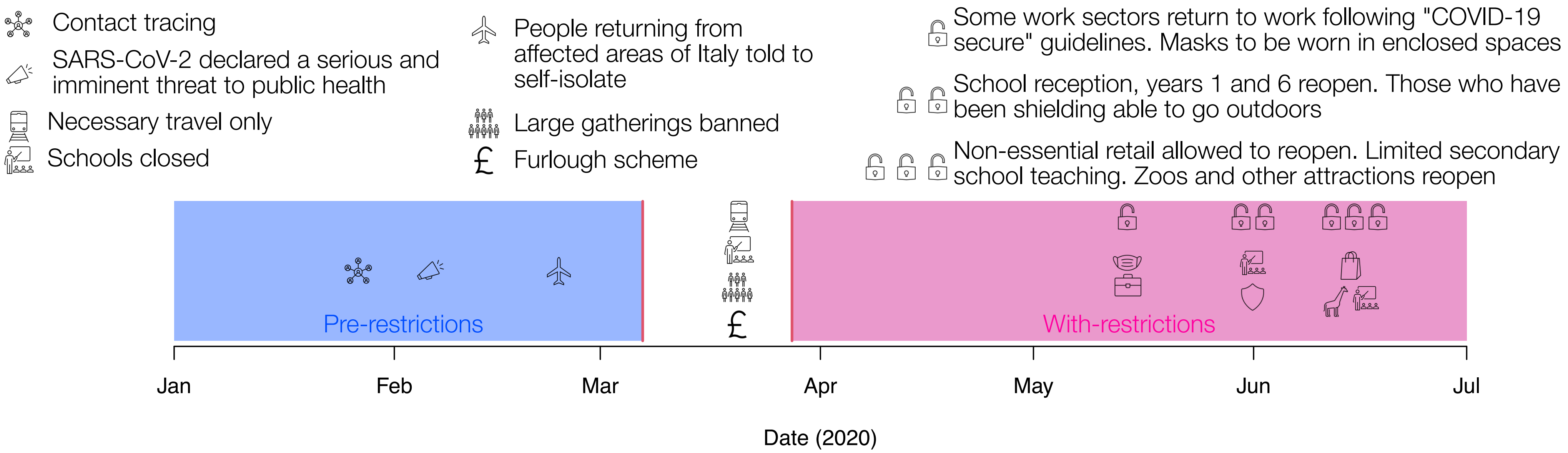

References

1 Scally G, Jacobson B, Abbasi K. The UK's public health response to covid-19. *The BMJ* 2020; **369**: 1–3.

2 Dunn P, Allen L, Cameron G, Alderwick H. COVID-19 policy tracker: A timeline of national policy and health system response to COVDI-19 in England. The Health Foundation. 2020. https://www.health.org.uk/news-and-comment/charts-and-infographics/covid-19-policy-tracker (accessed Dec 10, 2020).

Figure S2. Illustration of overall study population and condition-specific study populations (denominators).

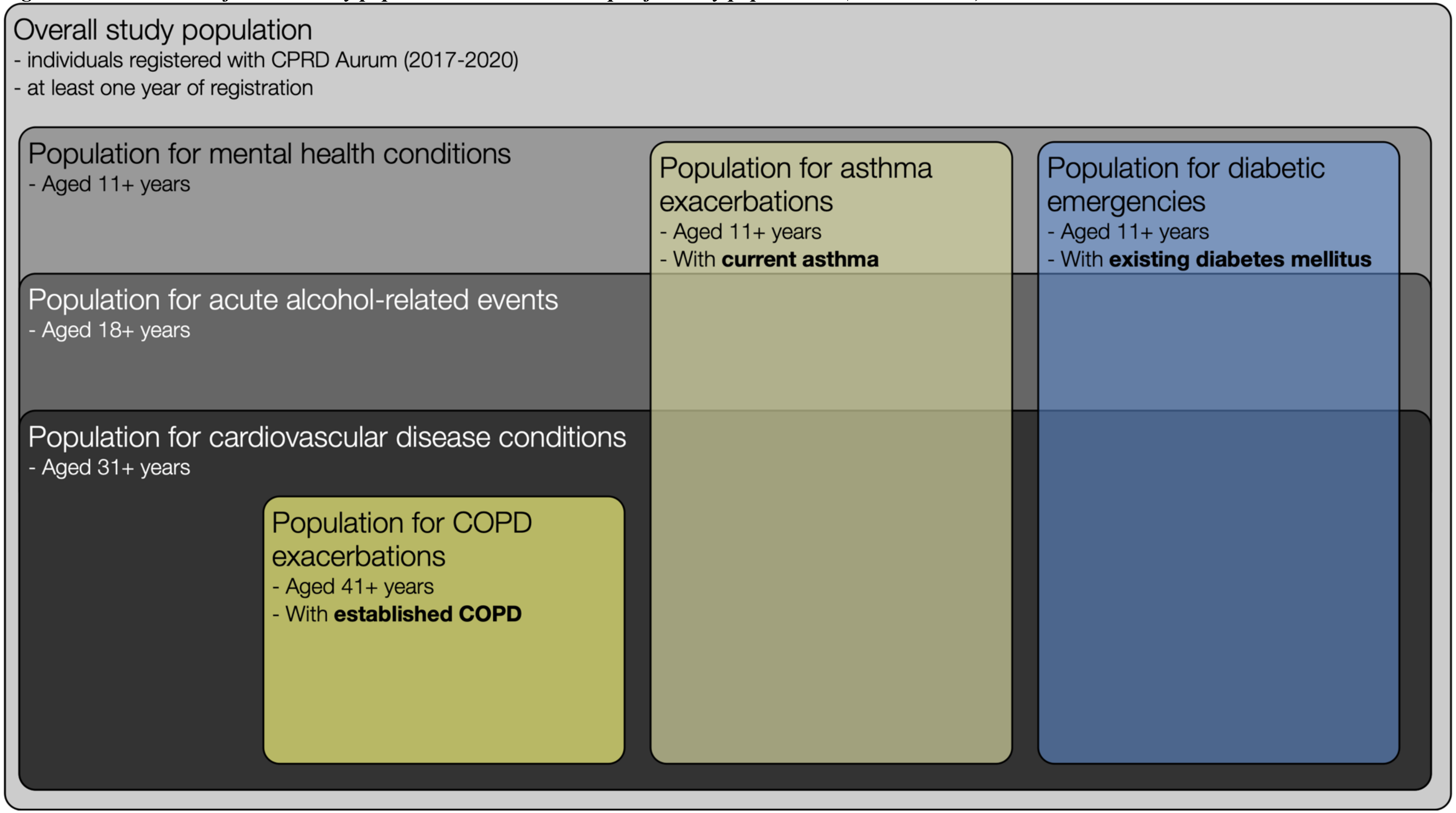

NB: Relative size of boxes do not represent relative sizes of study populations. For simplicity we have not shown the boxes for the study populations with asthma, COPD and diabetes overlapping, However, it is likely that there will be some individuals with multiple morbidities included in multiple populations, for example, there will be some people with both diabetes and COPD.

Abbreviations: COPD, chronic obstructive pulmonary disease; CPRD, Clinical Practice Research Datalink

Text S1

Definition of ethnicity and geographic region

We categorised ethnicity as White, South Asian, Black, Mixed or Other, based on a validated algorithm using routine recording in primary care records.[1] Ethnicity is recorded more completely in individuals registered with primary care practices in the UK from 2006 onwards (our study included data from 2017 onwards) when recording was incentivised by the introduction of remuneration for including ethnicity data in the Quality and Outcomes Framework.

We categorised geographic region into four broad categories based on the CPRD Aurum practice region variable: London, Midlands, North (covering: North East, North West, Yorkshire, Northern Ireland), and South (covering: South West, South Central, South East).[2] Northern Irish practices only started contributing to CPRD Aurum from 2019.

References

1 Mathur R, Bhaskaran K, Chaturvedi N, et al. Completeness and usability of ethnicity data in UK-based primary care and hospital databases. Journal of public health (Oxford, England) 2013; 36: 684–92.

2 Wolf A, Dedman D, Campbell J, et al. Data resource profile: Clinical Practice Research Datalink (CPRD) Aurum. International Journal of Epidemiology 2019; : 1–8.

Condition-specific denominator populations

Table S1 – Description of the denominator population for acute alcohol-related events, as measured in the first weeks of January 2017-2020. Percentages of total denominator population are shown in parentheses.

	Category	2017		2018		2019		2020	
	**Overall denominator**	**8,974,499**	**(100)**	**9,195,503**	**(100)**	**9,329,369**	**(100)**	**9,264,471**	**(100)**
**Age (years)**	18 - 20	343,983	(4)	354,773	(4)	362,880	(4)	362,944	(4)
	21 - 30	1,455,550	(16)	1,499,066	(16)	1,517,439	(16)	1,505,172	(16)
	31 - 40	1,559,933	(17)	1,622,838	(18)	1,662,883	(18)	1,661,724	(18)
	41 - 50	1,577,507	(18)	1,579,296	(17)	1,573,889	(17)	1,550,104	(17)
	51 - 60	1,520,720	(17)	1,564,290	(17)	1,590,738	(17)	1,580,348	(17)
	61 - 70	1,165,390	(13)	1,166,078	(13)	1,176,134	(13)	1,164,688	(13)
	71 - 80	833,570	(9)	881,099	(10)	907,289	(10)	904,486	(10)
	81 - 90	426,769	(5)	436,646	(5)	445,112	(5)	442,098	(5)
	91 - 100	91,077	(1)	91,417	(1)	93,005	(1)	92,907	(1)
**Ethnicity**	White	4,513,594	(50)	4,624,221	(50)	4,697,089	(50)	4,595,278	(50)
	South Asian	383,479	(4)	404,874	(4)	413,318	(4)	426,361	(5)
	Black	232,746	(3)	242,300	(3)	243,346	(3)	248,200	(3)
	Other	136,663	(2)	150,460	(2)	163,184	(2)	173,055	(2)
	Mixed	80,430	(1)	86,359	(1)	90,944	(1)	94,377	(1)
	Missing	3,627,587	(40)	3,687,289	(40)	3,721,488	(40)	3,727,200	(40)
**Sex**	Female	4,489,298	(50)	4,593,678	(50)	4,658,434	(50)	4,621,873	(50)
	Male	4,485,201	(50)	4,601,825	(50)	4,670,935	(50)	4,642,598	(50)
**Region**	North East	315,629	(4)	319,354	(4)	323,973	(4)	313,763	(3)
	North West	1,539,272	(17)	1,567,260	(17)	1,592,118	(17)	1,602,856	(17)
	Yorkshire and the Humber	341,192	(4)	349,911	(4)	357,458	(4)	329,935	(4)
	East Midlands	238,915	(3)	246,463	(3)	255,619	(3)	214,920	(2)
	West Midlands	1,427,095	(16)	1,452,751	(16)	1,450,408	(16)	1,469,456	(16)
	East of England	419,707	(5)	425,830	(5)	424,747	(5)	388,164	(4)
	South West	1,083,665	(12)	1,110,448	(12)	1,110,511	(12)	1,097,636	(12)
	South Central	1,126,410	(13)	1,150,518	(13)	1,164,597	(13)	1,175,184	(13)
	London	1,672,917	(19)	1,749,909	(19)	1,806,520	(19)	1,833,233	(20)
	South East Coast	752,133	(8)	764,640	(8)	785,323	(8)	780,288	(8)
	Northern Ireland	41,268	(1)	42,127	(1)	42,983	(1)	43,913	(1)

Table S2 – Description of the denominator population for asthma exacerbations, as measured in the first weeks of January 2017-2020. Percentages of total denominator population are shown in parentheses.

	Category	2017		2018		2019		2020	
**Overall**	Overall denominator	882,141	(100)	911,579	(100)	930,478	(100)	925,795	(100)
**Age (years)**	11 - 20	126,046	(14)	131,451	(14)	134,201	(14)	132,958	(14)
	21 - 30	121,166	(14)	126,651	(14)	130,488	(14)	131,277	(14)
	31 - 40	130,758	(15)	135,257	(15)	137,348	(15)	135,432	(15)
	41 - 50	151,529	(17)	151,355	(17)	150,289	(16)	146,610	(16)
	51 - 60	142,438	(16)	148,077	(16)	151,898	(16)	151,681	(16)
	61 - 70	102,330	(12)	103,790	(11)	105,957	(11)	106,028	(12)
	71 - 80	69,801	(8)	74,576	(8)	77,525	(8)	78,023	(8)
	81 - 90	32,877	(4)	34,870	(4)	36,675	(4)	37,367	(4)
	91 - 100	5,196	(1)	5,552	(1)	6,097	(1)	6,419	(1)
**Ethnicity**	White	485,515	(55)	502,818	(55)	515,848	(55)	509,045	(55)
	South Asian	41,203	(5)	43,692	(5)	44,426	(5)	45,405	(5)
	Black	21,876	(3)	23,040	(3)	23,424	(3)	23,991	(3)
	Other	6,956	(1)	7,623	(1)	8,132	(1)	8,533	(1)
	Mixed	9,498	(1)	10,434	(1)	11,028	(1)	11,477	(1)
	Missing	317,093	(36)	323,972	(36)	327,620	(35)	327,344	(35)
**Sex**	Female	500,972	(57)	514,796	(57)	522,970	(56)	517,863	(56)
	Male	381,169	(43)	396,783	(44)	407,508	(44)	407,932	(44)
**Region**	North East	30,297	(3)	31,003	(3)	31,760	(3)	31,015	(3)
	North West	158,004	(18)	162,735	(18)	166,655	(18)	168,060	(18)
	Yorkshire and the Humber	32,373	(4)	33,480	(4)	34,379	(4)	31,978	(4)
	East Midlands	23,406	(3)	24,587	(3)	25,410	(3)	21,377	(2)
	West Midlands	144,115	(16)	148,309	(16)	149,439	(16)	152,152	(16)
	East of England	43,360	(5)	44,275	(5)	44,333	(5)	40,822	(4)
	South West	112,592	(13)	116,876	(13)	118,531	(13)	117,871	(13)
	South Central	115,881	(13)	119,356	(13)	121,633	(13)	122,852	(13)
	London	144,994	(16)	151,694	(17)	156,521	(17)	158,105	(17)
	South East Coast	71,168	(8)	73,186	(8)	75,748	(8)	75,400	(8)
	Northern Ireland	4,429	(1)	4,536	(1)	4,627	(1)	4,709	(1)

Table S3 – Description of the denominator population for COPD exacerbations, as measured in the first weeks of January 2017-2020. Percentages of total denominator population are shown in parentheses.

	Category	2017		2018		2019		2020	
**Overall**	Overall denominator	283,406	(100)	272,201	(100)	259,039	(100)	239,809	(100)
**Age (years)**	11 - 20	-	-	-	-	-	-	-	-
	21 - 30	-	-	-	-	-	-	-	-
	31 - 40	-	-	-	-	-	-	-	-
	41 - 50	14,445	(5)	12,271	(5)	10,312	(4)	8,349	(4)
	51 - 60	46,909	(17)	43,846	(16)	40,586	(16)	36,369	(15)
	61 - 70	83,332	(29)	77,256	(28)	71,914	(28)	65,626	(27)
	71 - 80	88,906	(31)	88,889	(33)	86,442	(33)	81,384	(34)
	81 - 90	43,636	(15)	43,637	(16)	43,304	(17)	41,648	(17)
	91 - 100	6,178	(2)	6,302	(2)	6,481	(3)	6,433	(3)
**Ethnicity**	White	182,453	(64)	175,964	(65)	167,920	(65)	154,549	(64)
	South Asian	3,048	(1)	3,037	(1)	2,942	(1)	2,851	(1)
	Black	2,086	(1)	2,033	(1)	1,915	(1)	1,843	(1)
	Other	766	(0)	759	(0)	754	(0)	731	(0)
	Mixed	767	(0)	767	(0)	726	(0)	689	(0)
	Missing	94,286	(33)	89,641	(33)	84,782	(33)	79,146	(33)
**Sex**	Female	132,967	(47)	128,166	(47)	122,419	(47)	113,825	(48)
	Male	150,439	(53)	144,035	(53)	136,620	(53)	125,984	(53)
**Region**	North East	14,877	(5)	14,154	(5)	13,498	(5)	12,298	(5)
	North West	60,924	(22)	58,435	(22)	55,797	(22)	52,519	(22)
	Yorkshire and the Humber	11,409	(4)	11,025	(4)	10,515	(4)	9,042	(4)
	East Midlands	6,905	(2)	6,601	(2)	6,353	(3)	4,717	(2)
	West Midlands	47,419	(17)	45,777	(17)	43,713	(17)	41,547	(17)
	East of England	12,366	(4)	11,776	(4)	10,897	(4)	9,247	(4)
	South West	37,036	(13)	35,626	(13)	33,180	(13)	30,647	(13)
	South Central	30,381	(11)	29,035	(11)	27,586	(11)	26,097	(11)
	London	36,756	(13)	35,505	(13)	34,057	(13)	31,941	(13)
	South East Coast	23,229	(8)	22,228	(8)	21,479	(8)	19,876	(8)
	Northern Ireland	1,585	(1)	1,538	(1)	1,492	(1)	1,428	(1)

Table S4 – Description of the denominator population for cardiovascular conditions, as measured in the first weeks of January 2017-2020. Percentages of total denominator population are shown in parentheses.

	Category	2017		2018		2019		2020	
**Overall**	Overall denominator	7,174,966	(100)	7,341,664	(100)	7,449,050	(100)	7,396,355	(100)
**Age**	11 - 20	-	-	-	-	-	-	-	-
	21 - 30	-	-	-	-	-	-	-	-
	31 - 40	1,559,933	(22)	1,622,838	(22)	1,662,883	(22)	1,661,724	(23)
	41 - 50	1,577,507	(22)	1,579,296	(22)	1,573,889	(21)	1,550,104	(21)
	51 - 60	1,520,720	(21)	1,564,290	(21)	1,590,738	(21)	1,580,348	(21)
	61 - 70	1,165,390	(16)	1,166,078	(16)	1,176,134	(16)	1,164,688	(16)
	71 - 80	833,570	(12)	881,099	(12)	907,289	(12)	904,486	(12)
	81 - 90	426,769	(6)	436,646	(6)	445,112	(6)	442,098	(6)
	91 - 100	91,077	(1)	91,417	(1)	93,005	(1)	92,907	(1)
**Ethnicity**	White	3,779,955	(53)	3,871,985	(53)	3,937,363	(53)	3,858,594	(52)
	South Asian	293,049	(4)	312,242	(4)	320,937	(4)	332,222	(5)
	Black	180,227	(3)	187,706	(3)	188,628	(3)	192,517	(3)
	Other	86,473	(1)	94,994	(1)	102,469	(1)	109,191	(2)
	Mixed	56,005	(1)	60,115	(1)	63,305	(1)	65,851	(1)
	Missing	2,779,257	(39)	2,814,622	(38)	2,836,348	(38)	2,837,980	(38)
**Sex**	Female	3,618,289	(50)	3,695,459	(50)	3,745,435	(50)	3,712,398	(50)
	Male	3,556,677	(50)	3,646,205	(50)	3,703,615	(50)	3,683,957	(50)
**Region**	North East	248,280	(4)	250,999	(3)	254,945	(3)	246,165	(3)
	North West	1,225,731	(17)	1,245,139	(17)	1,263,473	(17)	1,272,387	(17)
	Yorkshire and the Humber	259,709	(4)	265,218	(4)	269,219	(4)	245,151	(3)
	East Midlands	172,559	(2)	178,118	(2)	183,218	(3)	147,195	(2)
	West Midlands	1,157,537	(16)	1,177,622	(16)	1,178,910	(16)	1,195,451	(16)
	East of England	354,740	(5)	359,343	(5)	358,035	(5)	326,440	(4)
	South West	878,048	(12)	896,719	(12)	895,673	(12)	883,609	(12)
	South Central	910,667	(13)	928,185	(13)	939,790	(13)	950,188	(13)
	London	1,309,139	(18)	1,370,179	(19)	1,419,386	(19)	1,447,147	(20)
	South East Coast	613,558	(9)	624,457	(9)	640,857	(9)	636,441	(9)
	Northern Ireland	31,721	(0)	32,401	(0)	33,069	(0)	33,667	(1)

Table S5 – Description of the denominator population for diabetic emergencies, as measured in the first weeks of January 2017-2020. Percentages of total denominator population are shown in parentheses.

	**Category**	**2017**		**2018**		**2019**		**2020**	
**Overall**	Overall denominator	699,396	(100)	690,707	(100)	674,150	(100)	643,682	(100)
**Age**	11 - 20	6,009	(1)	5,884	(1)	5,671	(1)	5,247	(1)
	21 - 30	11,036	(2)	10,729	(2)	10,378	(2)	9,833	(2)
	31 - 40	28,004	(4)	26,651	(4)	24,929	(4)	22,850	(4)
	41 - 50	76,508	(11)	71,678	(10)	66,118	(10)	59,991	(9)
	51 - 60	143,037	(21)	139,862	(20)	134,782	(20)	127,329	(20)
	61 - 70	172,312	(25)	167,644	(24)	163,513	(24)	156,783	(24)
	71 - 80	160,657	(23)	163,421	(24)	162,095	(24)	156,375	(24)
	81 - 90	88,587	(13)	90,987	(13)	92,066	(14)	90,370	(14)
	91 - 100	13,246	(2)	13,851	(2)	14,598	(2)	14,904	(2)
**Ethnicity**	White	364,403	(52)	358,470	(52)	349,154	(52)	327,187	(51)
	South Asian	53,148	(8)	54,242	(8)	53,394	(8)	52,862	(8)
	Black	26,619	(4)	26,888	(4)	26,052	(4)	25,594	(4)
	Other	6,695	(1)	7,080	(1)	7,255	(1)	7,380	(1)
	Mixed	5,843	(1)	5,972	(1)	5,952	(1)	5,842	(1)
	Missing	242,688	(35)	238,055	(35)	232,343	(35)	224,817	(35)
**Sex**	Female	309,214	(44)	305,090	(44)	297,288	(44)	283,621	(44)
	Male	390,182	(56)	385,617	(56)	376,862	(56)	360,061	(56)
**Region**	North East	25,575	(4)	25,037	(4)	24,485	(4)	22,901	(4)
	North West	123,120	(18)	120,535	(18)	117,708	(18)	113,871	(18)
	Yorkshire And The Humber	25,341	(4)	25,025	(4)	24,454	(4)	21,353	(3)
	East Midlands	16,751	(2)	16,642	(2)	16,380	(2)	12,458	(2)
	West Midlands	124,167	(18)	121,841	(18)	116,894	(17)	114,183	(18)
	East of England	29,713	(4)	29,148	(4)	27,858	(4)	23,943	(4)
	South West	83,235	(12)	82,172	(12)	78,483	(12)	74,385	(12)
	South Central	78,777	(11)	77,553	(11)	75,859	(11)	73,981	(12)
	London	133,720	(19)	134,642	(20)	134,380	(20)	131,521	(20)
	South East Coast	54,599	(8)	53,806	(8)	53,501	(8)	51,053	(8)
	Northern Ireland	2,831	(0)	2,789	(0)	2,725	(0)	2,653	(0)

Trends for each condition by stratification variables

Figure S3 – Stratified by ethnicity

Percentage of study populations with primary care contacts for each health condition over 2020, by ethnicity. Boxplots show the historical average (median [IQR]) percentage of study population with GP contacts for the condition of interest. Coloured lines, weekly percentage of eligible population with GP contacts for the condition of interest in 2020. Red dotted line, introduction of restrictions in UK on March 23rd 2020. If ethnicity information was missing then data are not shown. Note that cell counts with fewer than five contacts in one week in 2020 have been suppressed.

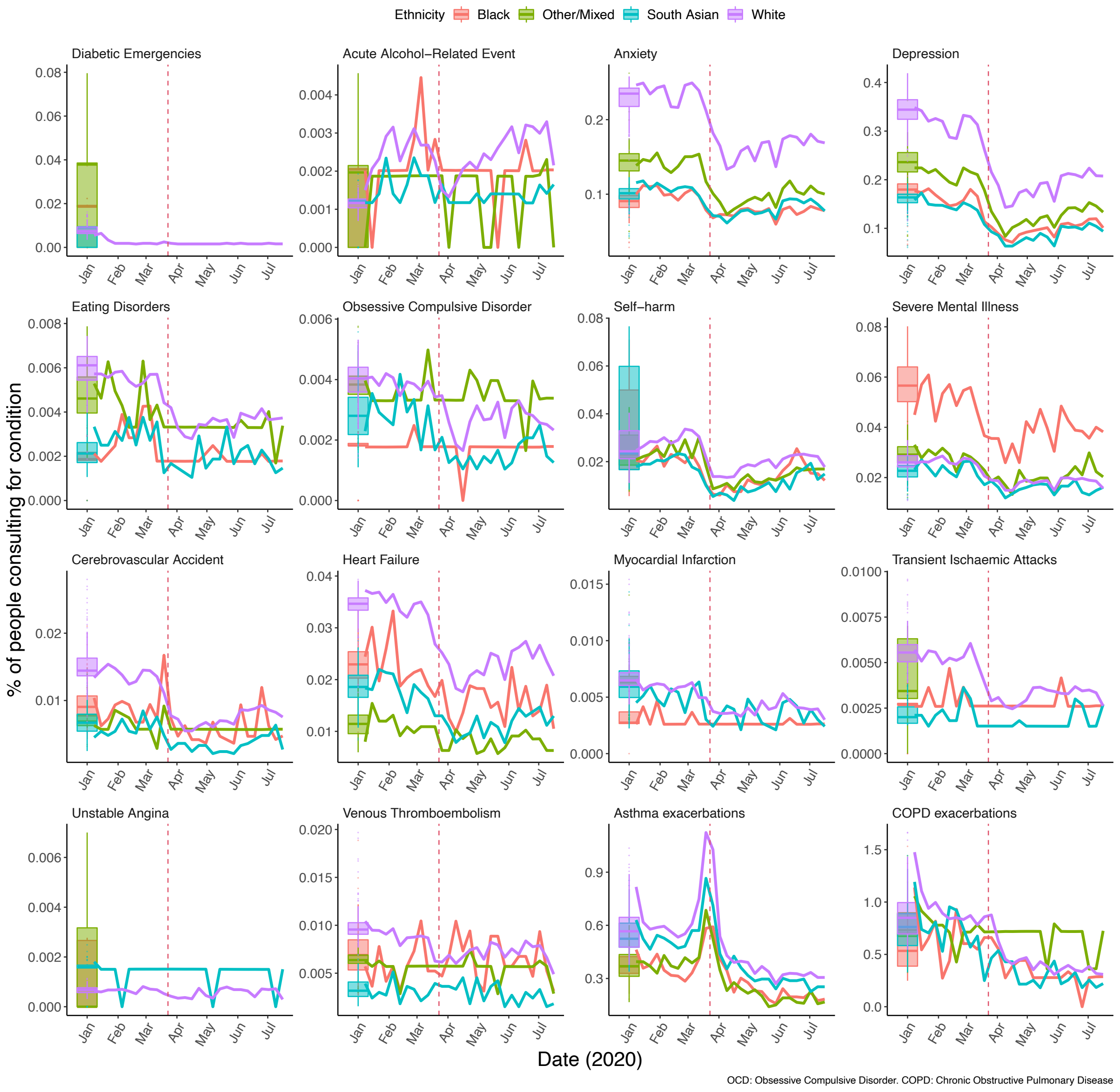

Figure S4 – Stratified by sex

Percentage of study populations with primary care contacts for each health condition over 2020, by sex. Boxplots show the historical average (median [IQR]) percentage of study populations with GP contancts for the condition of interest. Coloured lines, weekly percentage of eligible population that with GP contacts for the condition of interest in 2020. Red dotted line, introduction of restrictions in UK on March 23rd 2020. Note that cell counts with fewer than 5 outcomes in one week in 2020 have been suppressed.

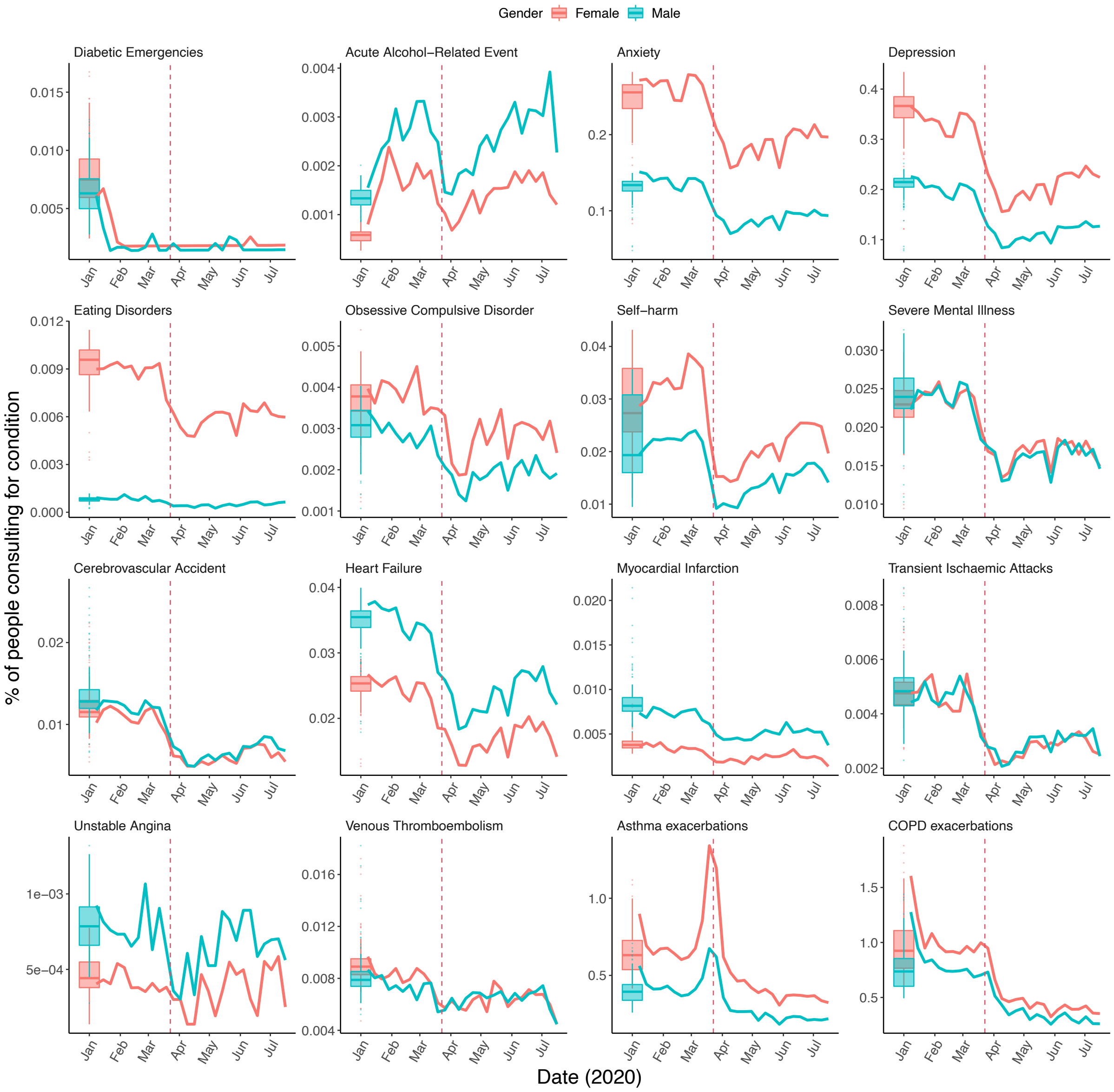

OCD: Obsessive Compulsive Disorder. COPD: Chronic Obstructive Pulmonary Disease

Figure S5 – Stratified by geographic region.
Percentage of study populations with GP contacts for each health condition over 2020, by region. Boxplots show the historical average (median [IQR]) percentage of study populations with GP contacts for the condition of interest. Coloured lines, weekly percentage of eligible population with GP contacts for the condition of interest in 2020. Red dotted line, introduction of restrictions in UK on March 23rd 2020. Data are not shown if information on region was missing and cell counts with fewer than five contacts in one week in 2020 have been suppressed.

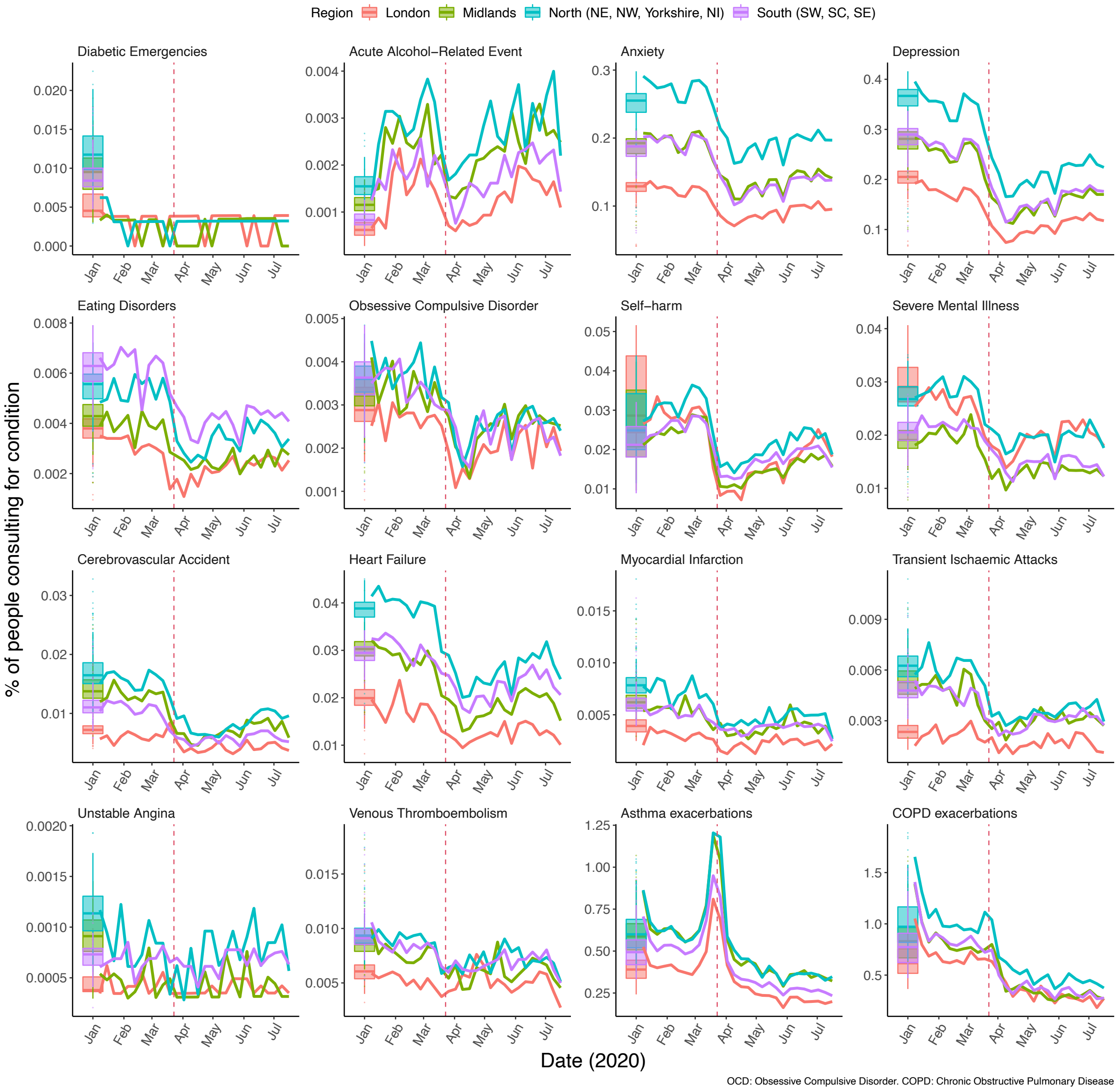

Text S2

ITS – the effect of inclusion of different length time series to define the pre-lockdown period

In our study protocol we planned to use data from 2017 to 2020 to conduct our interrupted time series analysis. We were able to use the full data for all conditions except self-harm, where the data showed a marked and instantaneous level shift in March 2018 (**Figure S6**). Since we hypothesised that this change in recording was likely to be related to a change in primary care coding practice and not reflective of underlying disease burden, these data were excluded from our analysis for self-harm. If we had included this data in the analysis for the definition of pre-restrictions, it would have led to an overestimate of the contact rate for self-harm in March 2020 and therefore overestimated the effect of the restrictions on self-harm consultation. For completeness we present the analysis with data constrained to 2019 onwards for all conditions (**Figure S7**) and the estimates for the effect of the restictions in the immediate reduction in primary care contacts and recovery of contact rates following the introduction of restrictions are shown in a forest plot (**Figure S8**). Pre-lockdown was defined as the period up until 7th March and three weeks of data (March 8th to March 28th 2020) were excluded, as in the main paper.

Figure S6 – As Figure 3, full data series plotted. Pre-lockdown period defined as 2017 until 7th March 2020 for all conditions (including self-harm; main analysis excluded 2017-2018 data for self-harm).
Data excluded for 3 weeks between pre- and post-introduction of restriction periods (March 8th to March 28th 2020 inclusive). Odds ratios in B show the relative change in the log odds of contacts for a particular condition for the week commencing 29th March 2020 compared to the week commencing 1st March 2020.

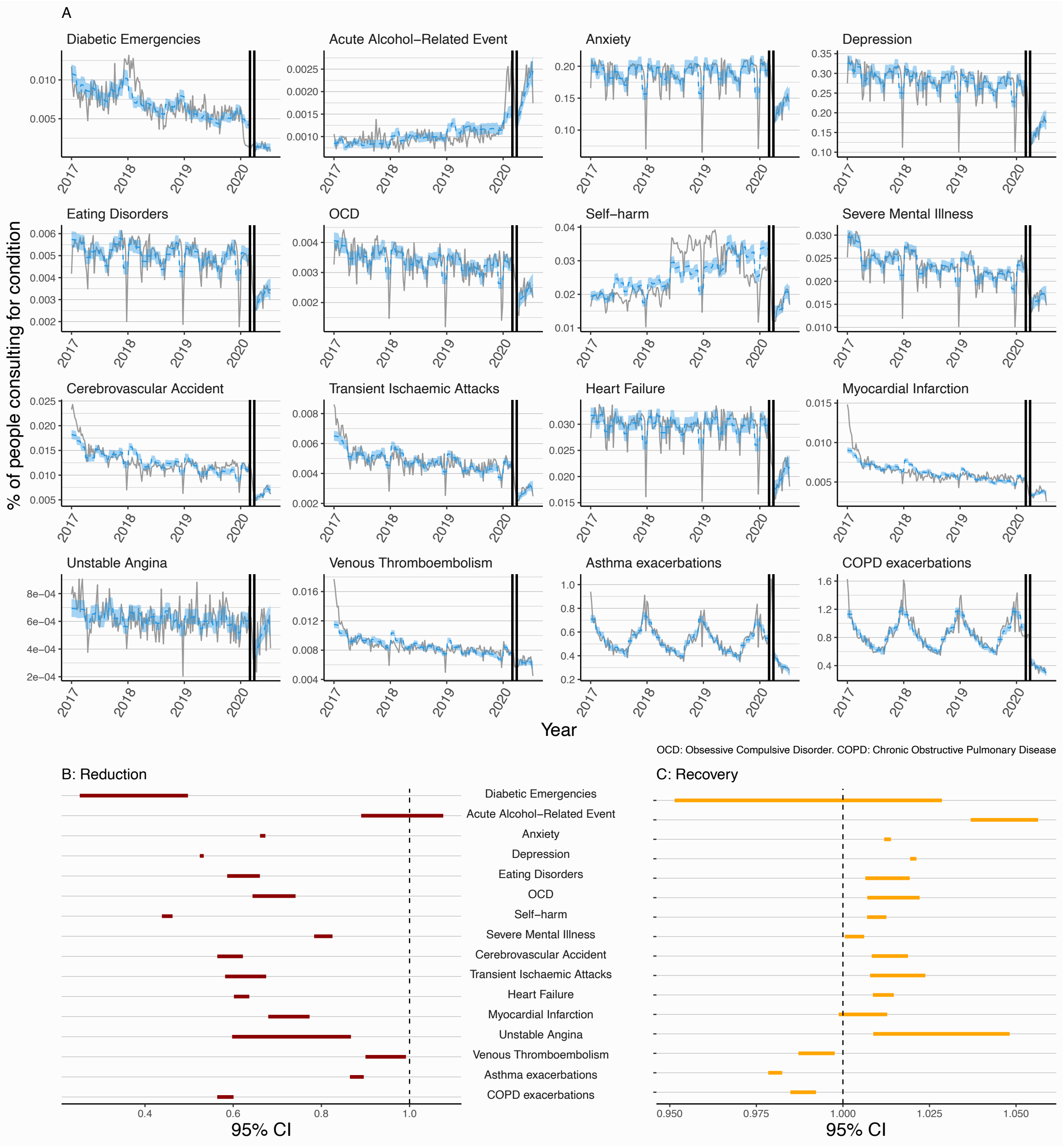

Figure S7 – As Figure 3, full data series plotted. Pre-lockdown period defined as 2019 until 7th March 2020 for all conditions.

Data excluded for 3 weeks between pre-lockdown and with restrictions periods (i.e., March 8th to March 28th 2020 inclusive). Odds ratios in B show the relative change in the log odds of contacts for a particular condition in the week commencing 29th March 2020 compared to the week commencing 1st March 2020.

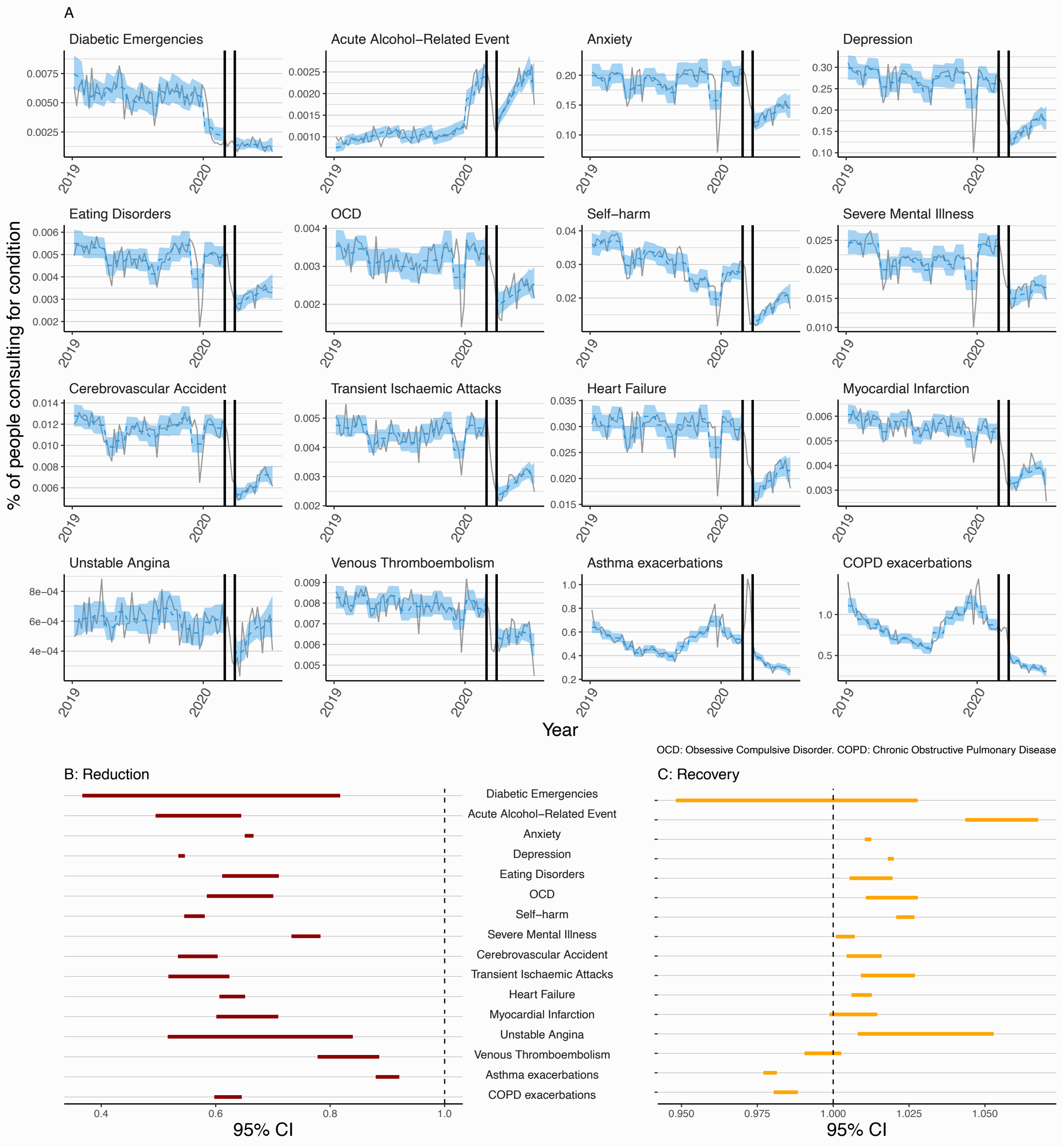

Figure S8 – A forest plot showing the effect of using different data periods on the estimated effect of the introduction of restrictions on primary care contact behaviour.
The plot "reduction" shows the odds ratio for the intervention (introduction of restrictions) in our ITS, this shows the relative change in contacts between the week commencing 29th March and the week commencing 1st March 2020. "Recovery" shows the effect of time on the odds of contacts in the post-introduction of restrictions period (i.e., from 29th March 2020). Colours indicate analyses using either data from 2017 or data from 2019 as the start of the pre-lockdown period (pre-lockdown defined as dates up to 7th March 2020).

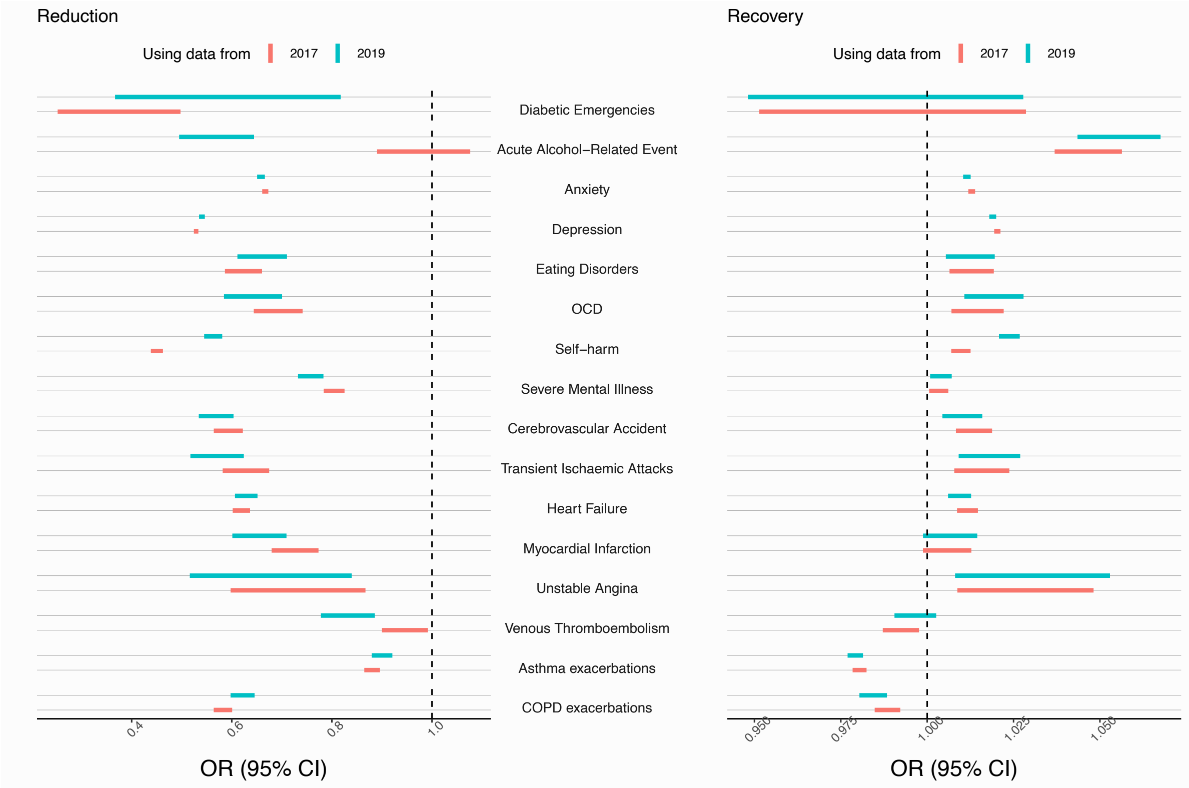

Text S3

Statistical analysis methods – additional information

We described all denominator study populations in the first week of January each year (2017-2020) (**Tables 1, S1-S5**). We plotted the percentage of our study populations with contacts for particular conditions in given weeks in 2020 and historical averages for that week (2017-2019). The historical average was the mean of the percentage of the study population consulting for a particular outcome in a given week between 2017 and 2019. We repeated this analysis stratified by age, region, ethnicity, and sex. To protect confidentiality, weekly cell counts were censored as 5 for any value between 0 and 5. If the total number of contacts for a particular condition in a particular strata never exceeded 5 in 2020 then data for these strata were not plotted in our analysis (**Figures S3-S5**).

ITS statistical model

We developed an interrupted time series (ITS) model to estimate the effect of restrictions on primary care contacts. The ITS model was a binomial generalised linear model for each condition at time t as defined below:

$$y_t = \beta_0 + \beta_1 T + \beta_2 R_t + \beta_3 T.R_t + \beta_4 Month_t + \beta_5 lagResid_t$$

Where y_t was the proportion of the eligible population with primary care contacts for the condition of interest (e.g. anxiety) in week t. The explanatory variables were: 1) time as a linear count of the week of the study (T); 2) COVID-19 restrictions, which was a binary variable set to 0 for the pre-lockdown period and 1 for the with-restrictions period (R_t); and 3) an interaction between these terms ($T.R_t$). The estimate for the coefficient β_2 is the estimate of the "reduction" in contacts between pre-lockdown and with-restriction periods. The estimate for the coefficient β_3 is the estimate of the "recovery" in contacts over time with-restrictions, i.e. the effect of a week increase in the with-restrictions period on the log odds of contact for the condition of interest.

The remaining two variables were the calendar month as a categorical variable ($Month_t$) to partially adjust for seasonal variation in primary care contacts. This adjustment was essential for several conditions that show a pronounced seasonal pattern in contact behaviour in our study (**Figure 2**). The final variable is the lagged residuals from the model ($lagResid_t$). The model was run without this variable and residuals from this reduced model were stored and lagged. These lagged residuals were then used as an explanatory variable in the full model. This was done to adjust for some of the autocorrelation that was present in all our time series, the value of y_t was dependent on the value of y_{t-1}.

To display outputs from this model we converted the predicted values (which were predicted log-odds of contact) to a percentage and calculated the 95% confidence interval on the scale of the linear predictor and converted these to a 95% confidence interval for the odds of contacting primary care for a given condition ($\frac{e^x}{1+e^x}$) and multiplying by 100 to convert it to a percentage. We adjusted for overdispersion when calculating these predicted values because we could not assume that at each time-point our n_i Bernoulli trials were indepentally and identically distributed. To do this, we took the Pearson goodness of fit statistic for the full model and divided it by the degrees of freedom in the model. This was used as the dispersion parameter when estimating predicted values instead of the assumed value of 1 from a binomial generalised linear model.

Finally, the structure of the ITS model to estimate the absolute effect of the restrictions on primary care contacts (**Table 3**) was identical except a Poisson model was used and the dynamic population size was included as an offset term. Predicted values from this model were similarly converted from the linear predictor scale (log count) to a count of the number of expected contacts with 95% confidence intervals for a population of 1 million people, which was the exponential of the predicted value for time t divided by the denominator population at time t and multiplied by 1 million.

Text S4

ITS with variable lockdown periods

To test the sensitivity of our findings to the choice of pre- and with-restrictions period we repeated the analysis with variable dates for the start of lockdown, and variable lengths of data exclusion to account for adjustment to lockdown in primary care contact behaviour. We varied the beginning of the adjustment-to-restrictions period beween 8th March (main analysis) and 22nd March 2020 (the week including the lockdown announcement in the UK). We varied the period of adjustment-to-restrictions (and therefore excluded data) between 0, 3, 5 or 7 weeks.

Table S6 – Reduction in contacts: Sensitivity analyses with variable adjustment-to-restrictions periods on primary care contacts comparing periods pre-lockdown and with restrictions

Results from a sensitivity analysis of variable adjustment-to-restriction periods on the relative change in GP contact behaviour for each health condition. These odds ratios measure the relative change in the log odds of a GP contact with a given health condition between the first week of the with-restrictions period compared to the last week of the pre-lockdown period.

**Start of behaviour change due to pandemic (i.e., start of the adjustment-to-restrictions period)**	**March 8th**			**March 22nd**			
**Duration of adjustment-to-restrictions period excluded from analysis** (all dates are inclusive)	**3 weeks (as in Figure 3B, i.e. main analysis)** (8 Mar to 28 Mar)	**5 weeks** (8 Mar to 11 Apr)	**7 weeks** (8 Mar to 25 Apr)	**3 weeks** (22 Mar to 11 Apr)	**5 weeks** (22 Mar to 25 Apr)	**7 weeks** (22 Mar to 9 May)	**0 weeks** (no data excluded, compared pre-lockdown [up to Mar 21] to with-restrictions [from Mar 22])
Diabetic Emergencies	0.35 (0.25-0.5)	0.41 (0.29-0.58)	0.42 (0.29-0.61)	0.43 (0.3-0.61)	0.44 (0.3-0.64)	0.49 (0.33-0.72)	0.38 (0.27-0.53)
Acute Alcohol-Related Events	0.98 (0.89-1.1)	1.2 (1.1-1.3)	1.3 (1.2-1.4)	1.3 (1.2-1.5)	1.4 (1.3-1.6)	1.6 (1.4-1.8)	1.2 (1.1-1.3)
Anxiety	0.67 (0.66-0.67)	0.69 (0.68-0.7)	0.72 (0.71-0.73)	0.7 (0.69-0.7)	0.73 (0.72-0.73)	0.74 (0.73-0.75)	0.68 (0.68-0.69)
Depression	0.53 (0.52-0.53)	0.55 (0.55-0.56)	0.59 (0.59-0.6)	0.56 (0.55-0.56)	0.6 (0.59-0.6)	0.63 (0.62-0.63)	0.55 (0.54-0.55)
Eating Disorders	0.62 (0.59-0.66)	0.67 (0.63-0.71)	0.71 (0.67-0.76)	0.68 (0.64-0.73)	0.73 (0.68-0.78)	0.72 (0.67-0.77)	0.65 (0.61-0.69)
OCD	0.69 (0.64-0.74)	0.78 (0.72-0.84)	0.88 (0.81-0.95)	0.78 (0.73-0.84)	0.88 (0.82-0.95)	0.91 (0.84-0.99)	0.7 (0.65-0.75)
Self-harm	0.56 (0.54-0.58)	0.67 (0.65-0.69)	0.74 (0.71-0.77)	0.7 (0.68-0.72)	0.78 (0.75-0.8)	0.81 (0.78-0.84)	0.67 (0.65-0.7)
Severe Mental Illness	0.8 (0.78-0.83)	0.87 (0.85-0.9)	0.92 (0.9-0.95)	0.88 (0.86-0.91)	0.93 (0.9-0.96)	0.93 (0.9-0.96)	0.82 (0.8-0.84)
Cerebrovascular Accident	0.59 (0.56-0.62)	0.62 (0.59-0.65)	0.66 (0.63-0.7)	0.64 (0.61-0.68)	0.69 (0.66-0.73)	0.74 (0.7-0.79)	0.65 (0.62-0.68)
Transient Ischaemic Attack	0.63 (0.58-0.67)	0.7 (0.65-0.76)	0.77 (0.71-0.84)	0.73 (0.67-0.78)	0.81 (0.74-0.88)	0.86 (0.78-0.93)	0.67 (0.62-0.72)
Heart Failure	0.62 (0.6-0.64)	0.65 (0.63-0.67)	0.69 (0.67-0.71)	0.66 (0.64-0.68)	0.7 (0.68-0.72)	0.75 (0.73-0.78)	0.64 (0.62-0.66)
Myocardial Infarction	0.72 (0.68-0.77)	0.78 (0.73-0.84)	0.86 (0.8-0.92)	0.82 (0.77-0.88)	0.9 (0.84-0.97)	0.99 (0.92-1.1)	0.79 (0.74-0.84)
Unstable Angina	0.72 (0.6-0.87)	0.87 (0.72-1)	0.96 (0.79-1.2)	0.87 (0.72-1.1)	0.97 (0.79-1.2)	1.2 (0.97-1.4)	0.74 (0.61-0.89)
Venous Thromboembolism	0.94 (0.9-0.99)	1 (0.96-1.1)	1 (0.98-1.1)	1.1 (1-1.1)	1.1 (1-1.2)	1.1 (1.1-1.2)	1 (0.96-1.1)
Asthma exacerbations	0.88 (0.86-0.9)	0.79 (0.78-0.81)	0.75 (0.73-0.77)	0.73 (0.71-0.74)	0.68 (0.67-0.7)	0.66 (0.65-0.68)	0.75 (0.74-0.77)
COPD exacerbations	0.58 (0.56-0.6)	0.53 (0.51-0.55)	0.53 (0.51-0.55)	0.53 (0.51-0.55)	0.53 (0.51-0.55)	0.52 (0.5-0.54)	0.59 (0.57-0.6)

Table S7 – Recovery in contacts: Sensitivity analyses with variable adjustment-to-restrictions periods on primary care contacts comparing periods pre-lockdown and with restrictions
Results from a sensitivity analysis of variable adjustment-to-restrictions periods on the relative effect of time on consultation behaviour for several health conditions with-restrictions. These odds ratios measure the relative effect on the log odds of a GP contact with a given health condition for a unit increase in time (weekly increases) with restrictions.

**Start of behaviour change due to pandemic (i.e., start of adjustment-to-restrictions period)**	**March 8th**			**March 22nd**			
**Duration of adjustment-to-restrictions period excluded from analysis** (all dates are inclusive)	**3 weeks (as in Figure 3C, i.e. main analysis)** (8 Mar to 28 Mar)	**5 weeks** (8 Mar to 11 Apr)	**7 weeks** (8 Mar to 25 Apr)	**3 weeks** (22 Mar to 11 Apr)	**5 weeks** (22 Mar to 25 Apr)	**7 weeks** (22 Mar to 9 May)	**0 weeks** (no data excluded, compared pre-lockdown [up to Mar 21] to with-restrictions [from Mar 22])
Diabetic Emergencies	0.99 (0.95-1.03)	0.97 (0.93-1.02)	0.96 (0.9-1.02)	0.97 (0.92-1.02)	0.96 (0.9-1.02)	0.93 (0.86-1.01)	0.99 (0.95-1.03)
Acute Alcohol-Related Events	1.05 (1.04-1.06)	1.03 (1.02-1.04)	1.03 (1.02-1.04)	1.02 (1.01-1.03)	1.01 (1-1.03)	1 (0.98-1.02)	1.03 (1.02-1.04)
Anxiety	1.01 (1.01-1.01)	1.01 (1.01-1.01)	1.01 (1.01-1.01)	1.01 (1.01-1.01)	1.01 (1.01-1.01)	1.01 (1.01-1.01)	1.01 (1.01-1.01)
Depression	1.02 (1.02-1.02)	1.02 (1.02-1.02)	1.02 (1.02-1.02)	1.02 (1.02-1.02)	1.02 (1.01-1.02)	1.01 (1.01-1.02)	1.02 (1.02-1.02)
Eating Disorders	1.01 (1.01-1.02)	1.01 (1-1.02)	1 (0.99-1.01)	1.01 (1-1.01)	1 (0.99-1.01)	1 (0.99-1.01)	1.01 (1-1.02)
OCD	1.01 (1.01-1.02)	1 (1-1.01)	0.99 (0.98-1)	1 (1-1.01)	0.99 (0.98-1)	0.98 (0.97-1)	1.01 (1.01-1.02)
Self-harm	1.02 (1.02-1.03)	1.01 (1.01-1.02)	1 (1-1.01)	1.01 (1.01-1.01)	1 (1-1)	0.99 (0.99-1)	1.01 (1.01-1.02)
Severe Mental Illness	1 (1-1.01)	0.99 (0.99-1)	0.99 (0.98-0.99)	0.99 (0.99-1)	0.99 (0.98-0.99)	0.98 (0.98-0.99)	1 (1-1)
Cerebrovascular Accident	1.01 (1.01-1.02)	1.01 (1.01-1.02)	1.01 (1-1.02)	1.01 (1-1.01)	1 (0.99-1.01)	0.99 (0.98-1)	1.01 (1-1.01)
Transient Ischaemic Attacks	1.02 (1.01-1.02)	1.01 (1-1.02)	1 (0.98-1.01)	1 (0.99-1.01)	0.99 (0.98-1)	0.98 (0.96-1)	1.01 (1-1.02)
Heart Failure	1.01 (1.01-1.01)	1.01 (1.01-1.01)	1 (1-1.01)	1.01 (1-1.01)	1 (1-1.01)	0.99 (0.98-1)	1.01 (1.01-1.01)
Myocardial Infarction	1.01 (1-1.01)	1 (0.99-1.01)	0.99 (0.98-1)	0.99 (0.99-1)	0.98 (0.97-0.99)	0.96 (0.95-0.98)	1 (0.99-1.01)
Unstable Angina	1.03 (1.01-1.05)	1.01 (0.99-1.04)	1 (0.97-1.03)	1.01 (0.99-1.04)	1 (0.97-1.03)	0.97 (0.93-1.01)	1.03 (1.01-1.05)
Venous Thromboembolism	0.99 (0.99-1)	0.98 (0.98-0.99)	0.98 (0.97-0.98)	0.98 (0.97-0.99)	0.97 (0.96-0.98)	0.96 (0.95-0.97)	0.99 (0.98-0.99)
Asthma exacerbations	0.98 (0.98-0.98)	0.99 (0.99-0.99)	0.99 (0.99-1)	0.99 (0.99-1)	1 (1-1)	1 (1-1.01)	0.99 (0.99-0.99)
COPD	0.99 (0.98-0.99)	1 (0.99-1)	1 (0.99-1)	1 (0.99-1)	1 (0.99-1)	1 (0.99-1.01)	0.99 (0.98-0.99)

Figure S9 – As Figure 3. Pre-lockdown period defined as 2017 until 7th March 2020.
Data excluded for 3 weeks between pre-lockdown and with-restrictions periods (i.e., adjustment-to-restrictions period: March 8th to March 28th 2020 inclusive). Odds ratios in B show the relative change in the log odds of contact for a particular condition in the week commencing 29th March compared to the week commencing 1st March 2020. Note, this is the same as Figure 3 but is included here for comparison with results from our sensitivity analysis.

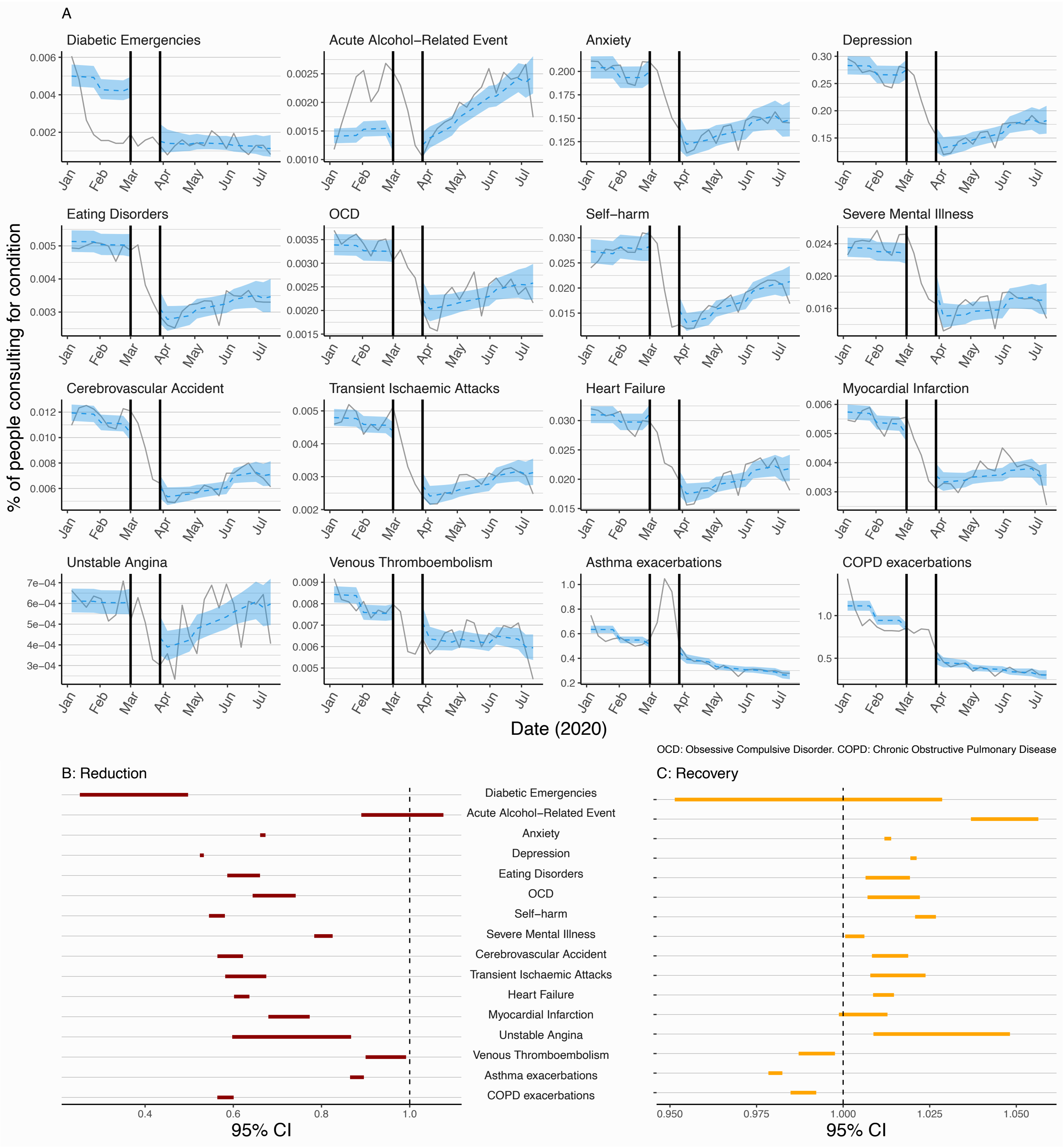

Figure S10 – As Figure 3. Pre-lockdown period defined as 2017 until 7th March 2020.
Data excluded for 5 weeks between pre-lockdown and with-restrictions periods (i.e., adjustment-to-restrictions period: March 8th to April 11th 2020 inclusive). Odds ratios in B show the relative change in the log odds of contact for a particular condition in the week commencing 12th April compared to the week commencing 1st March 2020.

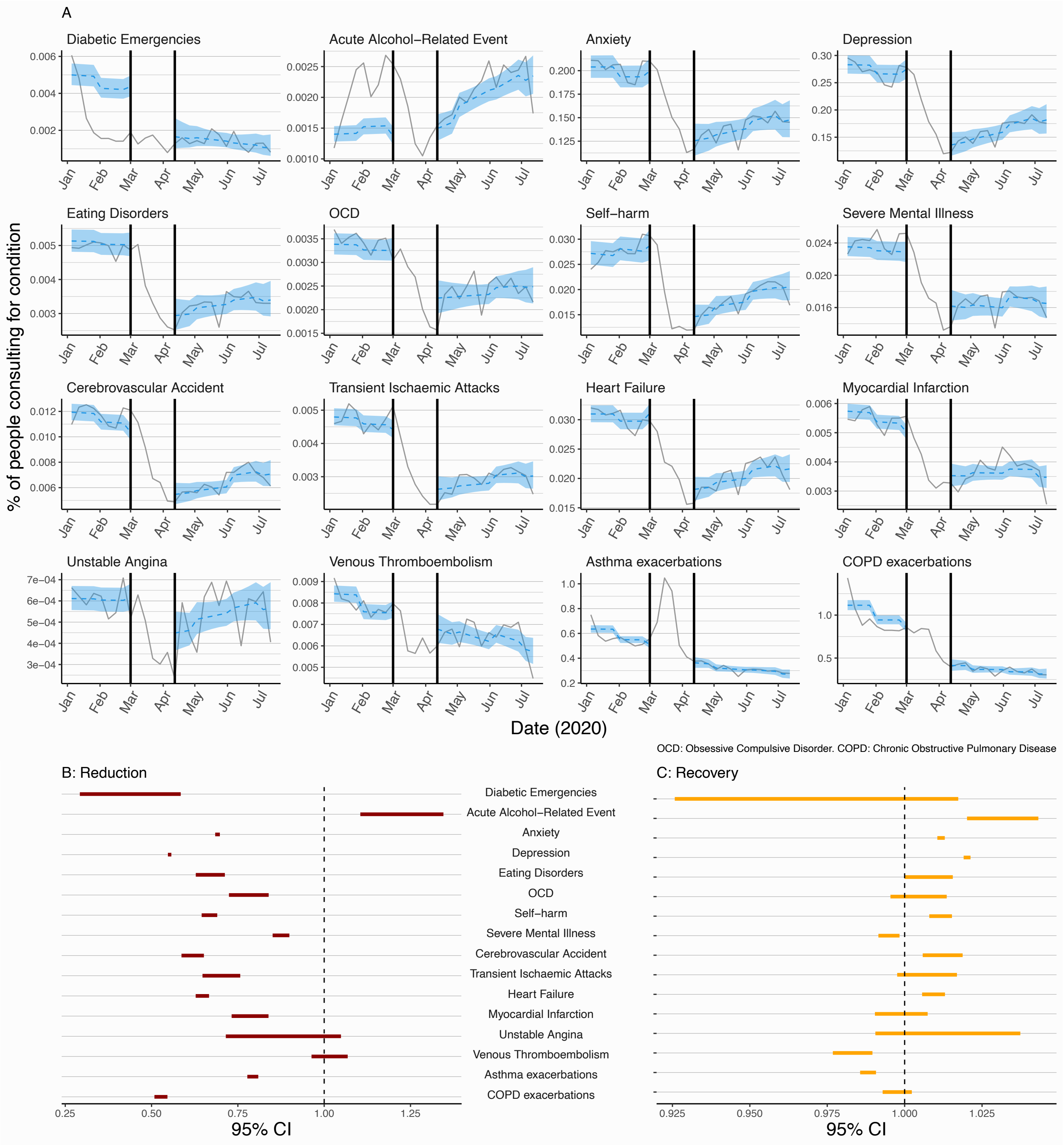

Figure S11 – As Figure 3. Pre-lockdown period defined as 2017 until 1st March 2020.
Data excluded for 7 weeks between pre-lockdown and with-restrictions periods (i.e., adjustment-to-restrictions period: March 8th to April 25th 2020 inclusive). Odds ratios in B show the relative change in the log odds of contact for a particular condition in the week commencing 26th April compared to the week commencing 1st March 2020.

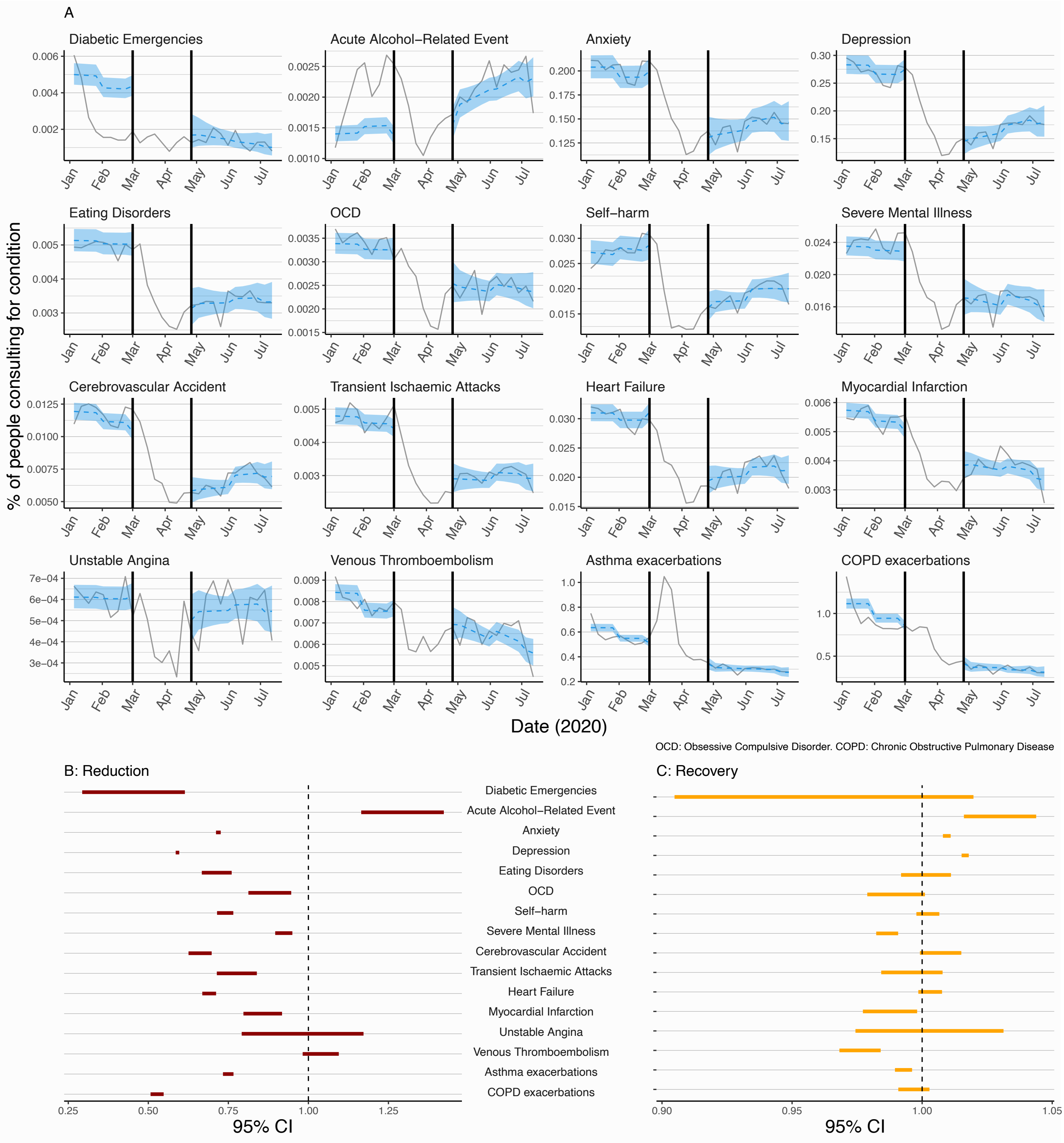

Figure S12 – As Figure 3. Pre-lockdown period defined as 2017 until 22nd March 2020.
Data excluded for 3 weeks between pre-lockdown and with-restrictions periods (i.e., adjustment-to-restrictions period: March 22nd to April 11th 2020 inclusive). Odds ratios in B show the relative change in the log odds of contact for a particular condition on the week commencing 12th April compared to the week commencing 15th March 2020.

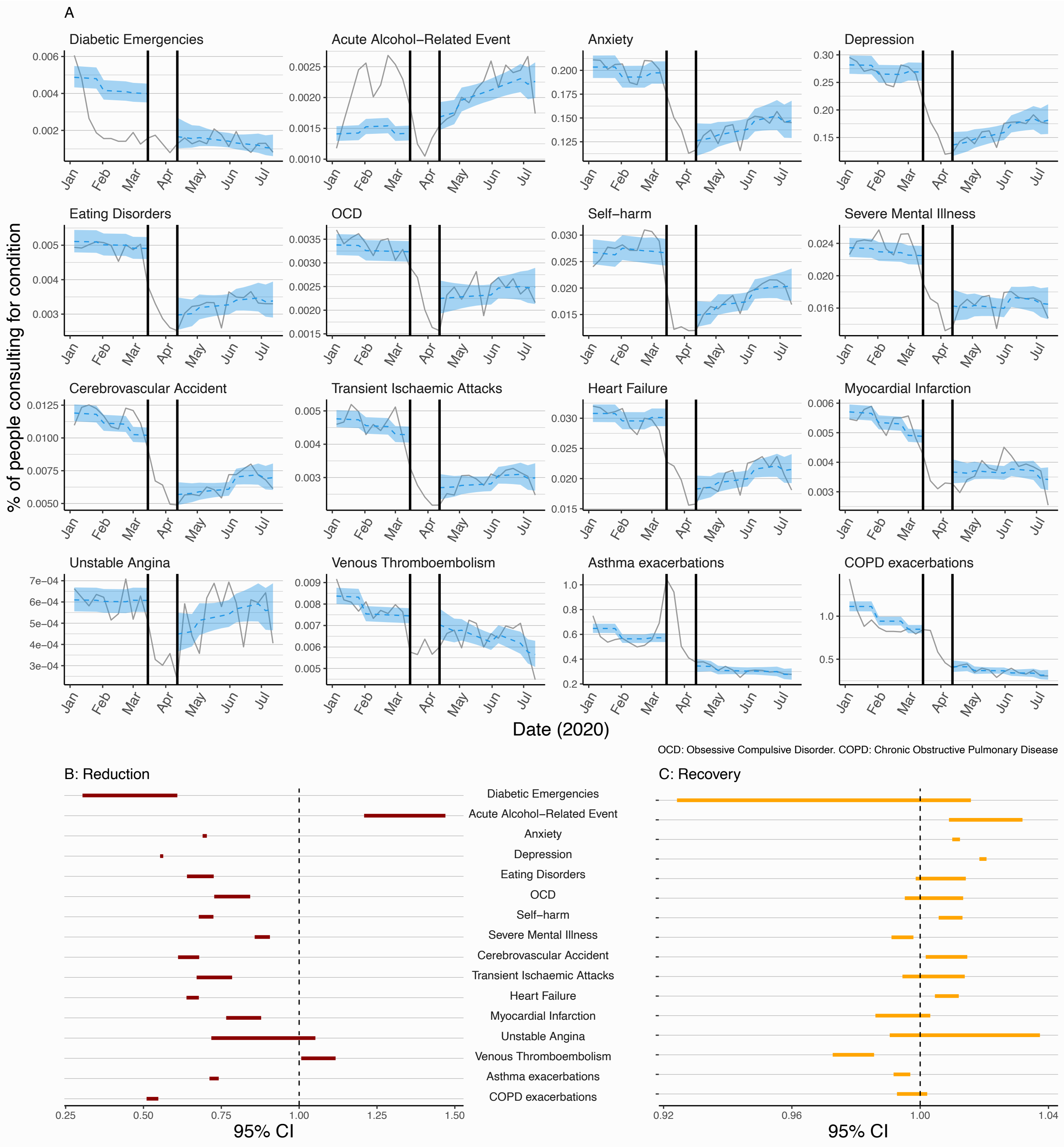

Figure S13 – As Figure 3. Pre-lockdown period defined as 2017 until 22nd March 2020.
Data excluded for 5 weeks between pre-lockdown and with-restrictions periods (i.e., adjustment-to-restrictions period: March 22nd to April 25th 2020 inclusive). Odds ratios in B show the relative change in the log odds of contact for a particular condition in the week commencing 26th April compared to the week commencing 15th March 2020.

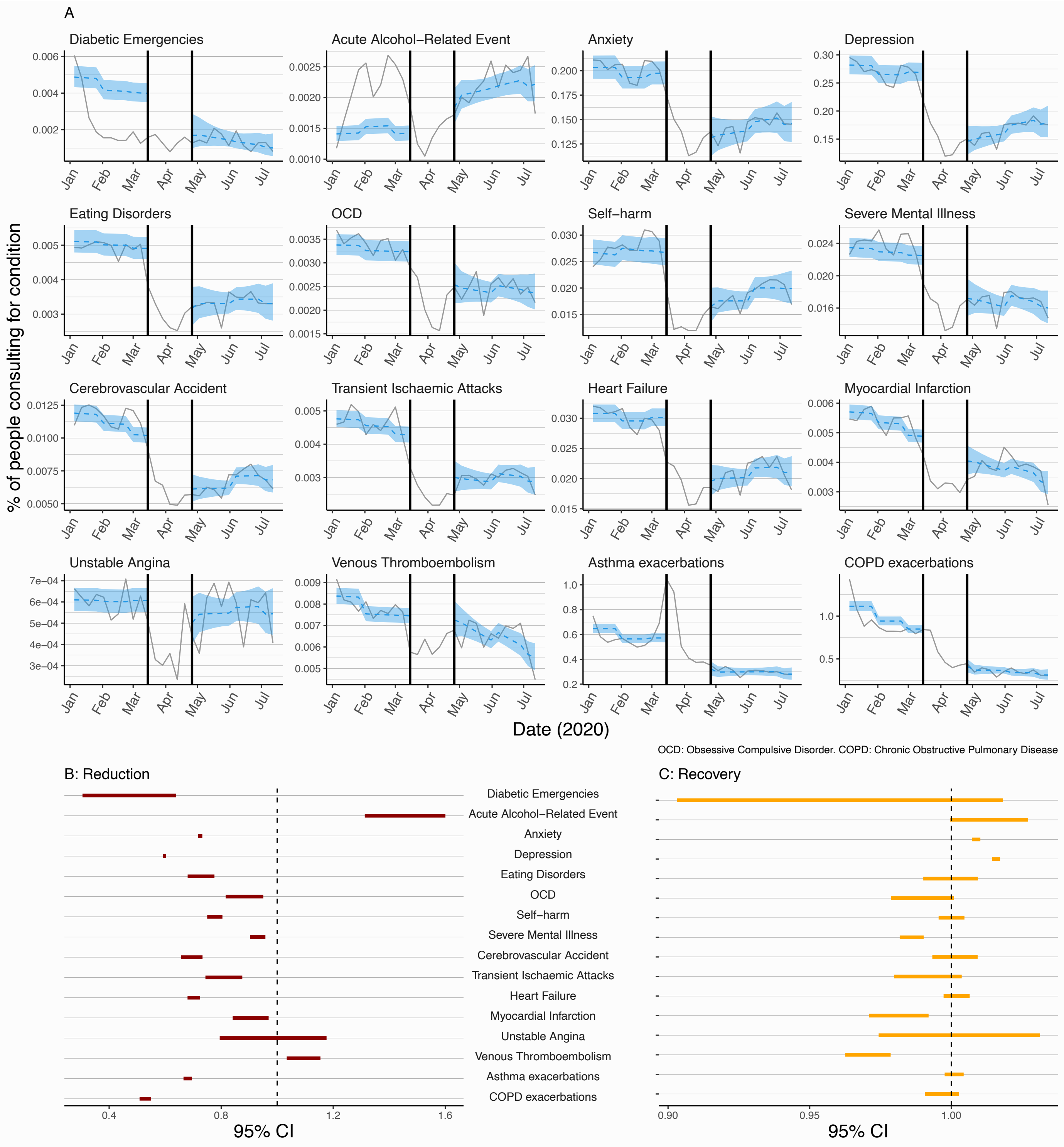

Figure S14 – As Figure 3. Pre-lockdown period defined as 2017 until 22nd March 2020.
Data excluded for 7 weeks between pre-lockdown and with-restrictions periods (i.e., adjustment-to-restrictions period: March 22nd to May 9th 2020 inclusive). Odds ratios in B show the relative change in the log odds of contact for a particular condition in the week commencing 10th May compared to the week commencing 15th March 2020.

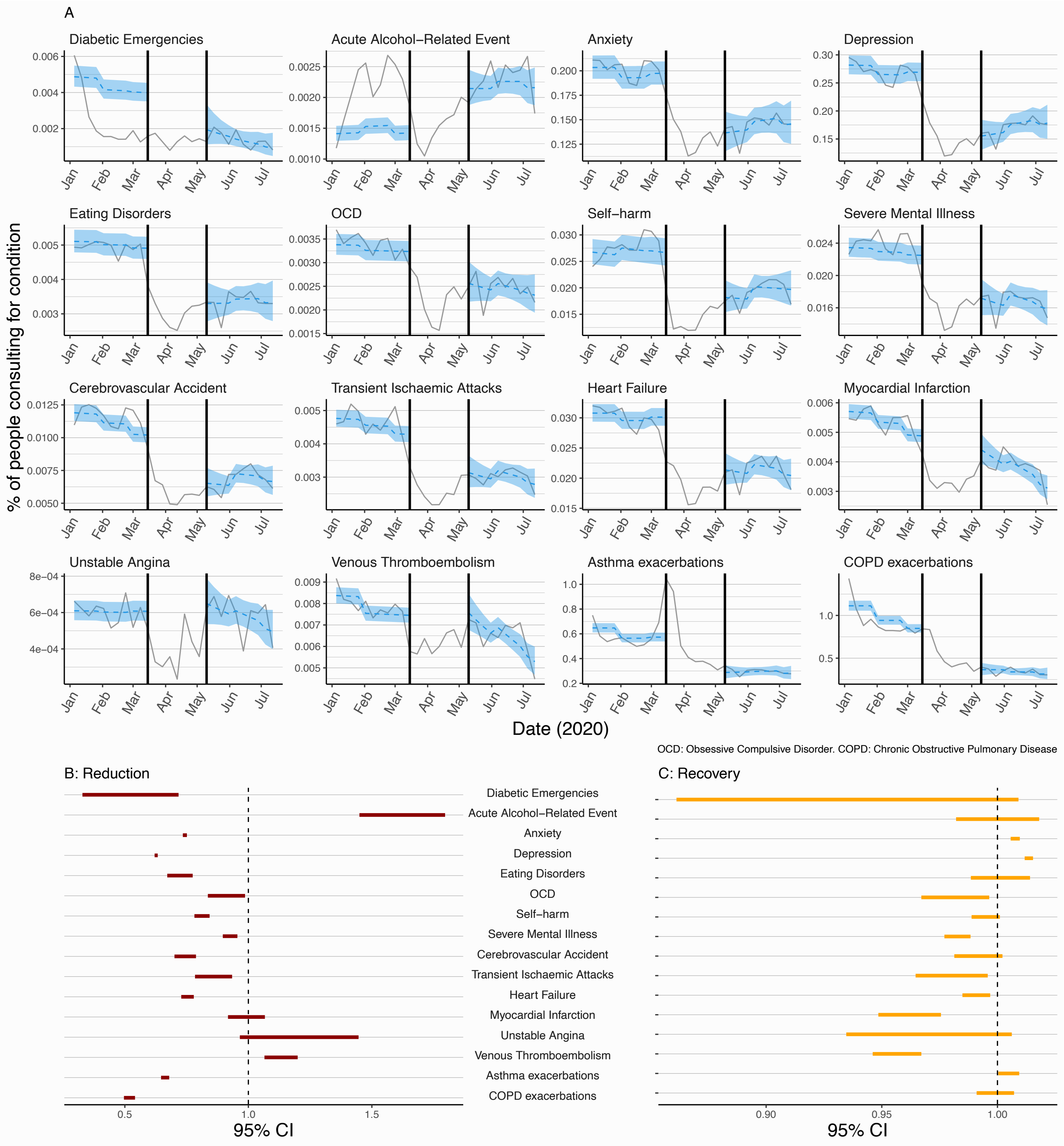

Figure S15 – As Figure 3 (i.e. main analysis). Pre-lockdown period defined as 2017 until 22nd March.
No data excluded (i.e., no adjustment-to-restrictions period), compared pre-lockdown (up to March 21st 2020) to with-restrictions (from March 22nd 2020). Odds ratios in B show the relative change in the log odds of contact for a particular condition on in the week commencing 29th March compared to the week commencing 22nd March 2020.

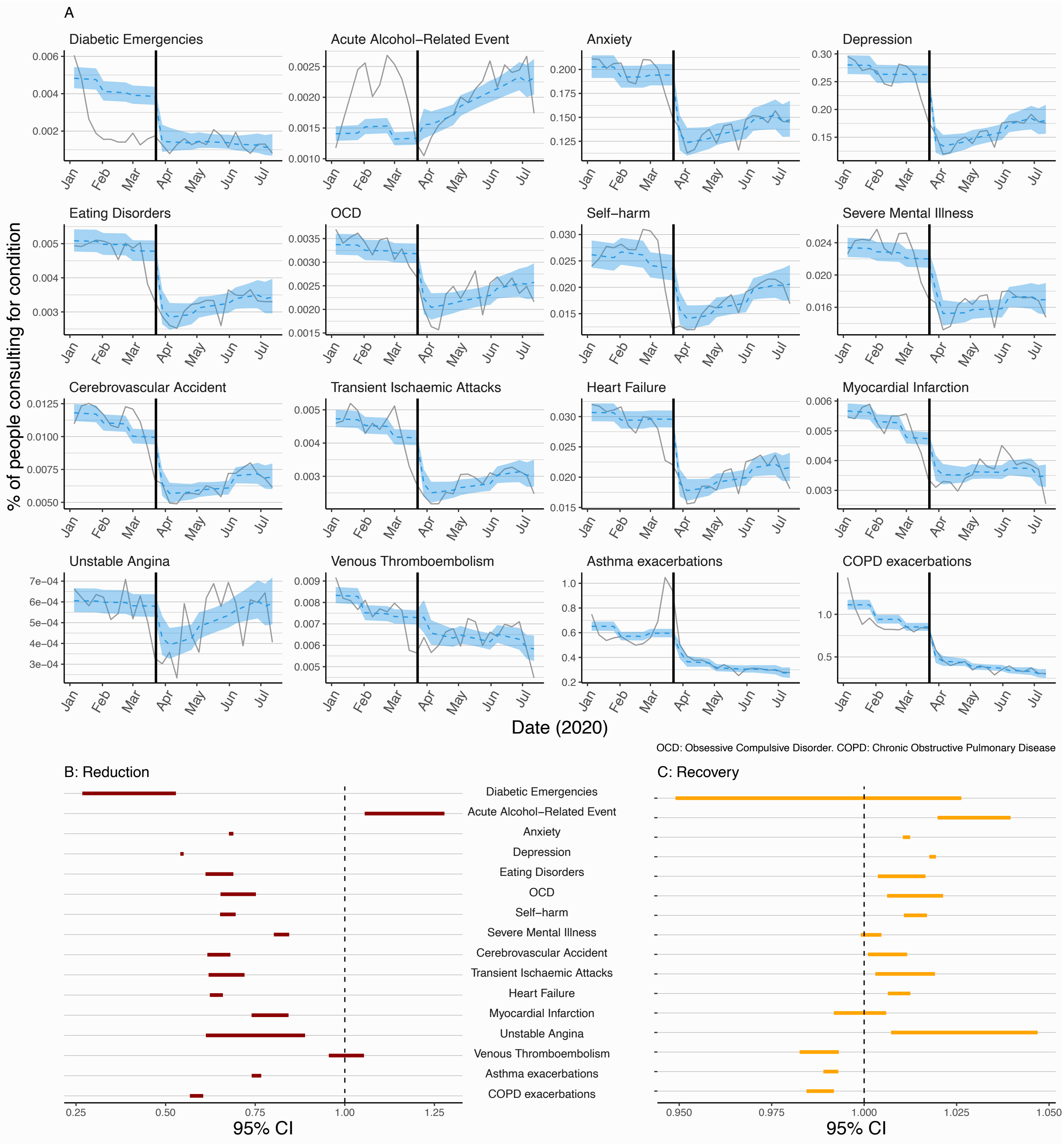

Text S5

Post-hoc sensitivity analysis: varying diabetic emergencies definition

Unlike other outcomes, we observed a decline in primary care contacts for diabetic emergencies at the start of 2020, before the implementation of a UK-wide restrictions in March 2020. This may be explained by natural variation, or be artefact due to the small number of conditions. Another explanation may be a delay in recording of hospital records of diabetic emergencies in primary care records (severe diabetic emergencies such as ketoacidoses are likely to lead to hospital admission) due to changes in working patterns in response to the restrictions, leading to inaccurate recording of the dates of contacts and consequently affecting the apparent distribution of contacts.

As a *post-hoc* sensitivity analysis we additionally included records for "non-diabetic hyperglycaemia" in our definition of "diabetic emergencies" (**Table S8**). People with diabetes mellitus were the denominator population for this condition, so it is likely that any hyperglycaemic records (regardless of whether they were labelled 'non-diabetic') were due to diabetes. **Figure S15** shows the results for the two definitions of diabetic emergency (i.e. main and sensitivity analyses) and indicates that even with the inclusion of "non-diabetic hyperglycaemia" as a diabetic emergency (**Figure S15B**) the percentage of diabetic emergency contacts is consistently lower than the historical average (except for spikes in two weeks of June and July 2020). Furthermore, when the definition includes "non-diabetic hyperglycaemia", there was a clear reduction in diabetic emergency contacts across 2020 compared to the 2017-2019 average. We also analysed the trend in all contacts for diabetes in 2020 (routine and emergency codes) (**Figure S15C**), which showed a rapid and sustained decrease beginning shortly before the introduction of UK-wide-restrictions in March 2020 compared to the historical weekly average between 2017-2019 (as is observed across the majority of other conditions).

Table S8. Main analysis and post-hoc sensitivity analyses with alternative definitions of diabetes condition.

Condition	Denominator population	Condition definition
**Diabetes**		
Diabetic emergencies (main analysis definition)	All individuals (aged ≥11 years) with prevalent diagnoses of diabetes mellitus at the start of each week of follow-up. Individuals contributed to the study population from the latest of the start of follow-up in the overall population and the date of their first record indicating a diagnosis of diabetes.	Any record of hyperglycaemia, hypoglycaemia, ketoacidosis, or diabetic coma. Multiple records occurring within **seven days** of each other were considered as representing the same event.
Diabetic emergencies (*post-hoc* sensitivity analysis)	As above	Any record of hyperglycaemia (recorded as "diabetic" or "non-diabetic"), hypoglycaemia, ketoacidosis, or diabetic coma. Multiple records occurring within **seven days** of each other were considered as representing the same event.
All diabetes primary care contacts (*post-hoc* sensitivity analysis)	As above	Any record of a consultation involving diabetes, routine or emergency. Multiple records occurring within **seven days** of each other were considered as representing the same event.

Figure S16 – Sensitivity analysis of the definition of diabetic contacts.
(A) trend of 2020 consultations for diabetic emergencies as defined in the main body of this paper.

(B) a post-hoc sensitivity analysis that classified "non-diabetic hyperglycaemia" as a diabetic emergency since it was recorded in a population of people with diabetes.

(C) a post-hoc sensitivity analysis of all diabetes consultations (emergency and routine) in 2020 compared to a historical average.

Black line, weekly historical average percentage of eligible population consulting (2017-2019, grey lines show the data for 2017, 2018, and 2019). Red line, weekly percentage of eligible percentage consulting in 2020. Red shaded region shows difference with historical average. Red dotted line, introduction of restrictions in UK on March 23rd.

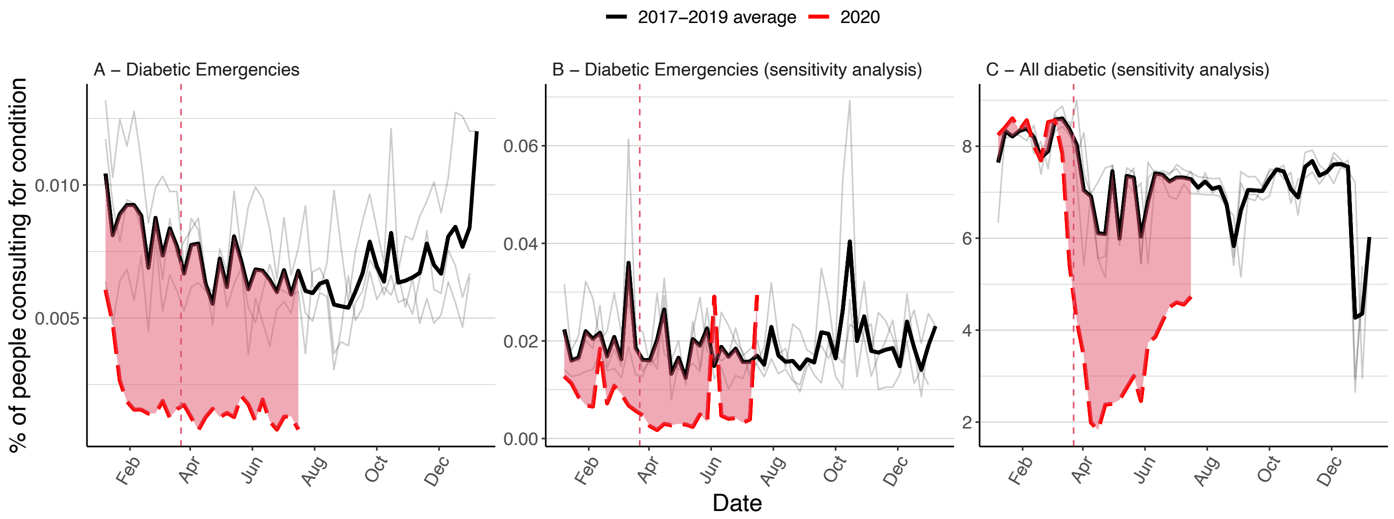

Study Protocol

**Applicants must complete all sections listed below<br>Applications with sections marked 'Not applicable' without justification will be returned as invalid**
**A. Study Title (Max. 255 characters, including spaces)**<br>Indirect acute effects of the COVID-19 pandemic on physical and mental health
**B. Lay Summary (Max. 250 words)**<br>We will investigate the effect of the COVID-19 pandemic on some key mental and physical health conditions not directly related to coronavirus infection. Understanding the indirect effects of the pandemic will inform UK healthcare policy by identifying population healthcare needs.<br><br>The COVID-19 pandemic has caused substantial illness and death. Much of the UK focus has been on pandemic management. However, the pandemic will have effects on wider mental and physical health, because of heightened distress and reduced healthcare resources for conditions other than COVID-19 (e.g. activities like monitoring and treatment of ongoing illnesses). People may also avoid seeking care for new and ongoing conditions due to anxiety about the pandemic (fear of infection, perceived burden on health service). Pandemic-related anxiety will also affect mental health, as will control measures to limit virus spread (e.g. self-isolation and employment worries).<br><br>We will look at specific diseases affected by different aspects of the pandemic: diabetic emergencies, lung diseases, heart disease emergencies, strokes, and mental illnesses, and compare patterns before and after lockdown measures. We will also explore whether the patterns of these specific diseases are different in different groups of people including people of different ages, men and women, different ethnicities, different levels of deprivation, and between people living in different regions, or rural and urban areas. This will help inform clinicians and health care service providers where healthcare resources are needed most.
**C. Technical Summary (Max. 300 words)**<br>We will analyse changes in key indirect mental and physical health outcomes, during and following the COVID-19 pandemic, including mental health outcomes (e.g. depression, anxiety, alcohol-related harms), and acute presentations of diabetes (e.g. ketoacidosis), respiratory (e.g. exacerbations of asthma and chronic obstructive pulmonary disease) and cardiovascular (e.g. myocardial infarction, unstable angina, stroke) diseases.<br><br>Initially, we will descriptively compare the proportions of weekly outcomes before (from January 2017) and after lockdown measures were introduced on 13th March 2020 (with sensitivity analyses looking at alternative time points). To calculate weekly outcome proportions, we will use different denominator populations depending on the outcome under investigation: 1) for acute diabetic and respiratory presentations, denominator populations will be individuals with existing diabetes (no age limits), asthma (aged 5years+), or chronic obstructive pulmonary disease (aged 40years+) as appropriate; and 2) for mental health outcomes (ages 5-17, and 18years+), alcohol-related harms (aged 18years+) and acute cardiovascular disease (aged 30years+), denominators will be the AURUM population from 2017 (restricted to specific ages depending on outcome).<br><br>Where possible, we will stratify the proportion of outcomes occurring each week by: age, sex, ethnicity, partnership, vulnerable status (i.e. individuals at particular risk of severe respiratory illness), socioeconomic deprivation, region, and urban/rural location. We will also explore outcome-specific stratification measures (e.g. long-term blood sugar control measures for diabetic emergencies).<br><br>We will aggregate data by week and strata, and make them available on our institutional website via an interactive data dashboard (supressing small event counts to preserve confidentiality).<br><br>We will then conduct a series of formal tests on specific hypotheses about changes in health burden following the pandemic. We will use generalised linear models and an interrupted time series design, where the interruption is defined at the date lockdown measures were introduced, and flexible functions of time control for pre-COVID temporal trends and seasonality.
**D. Outcomes to be Measured**<br>**Diabetes mellitus emergency presentations:** hyperglycaemia; hypoglycaemia, ketoacidosis; diabetic coma.<br><br>**Mental health outcomes:** anxiety; depression; eating disorders (anorexia; bulimia; others); fatal and non-fatal self-harm; obsessive-compulsive disorder (OCD); serious mental illness (i.e. schizophrenia, bipolar disorder and other psychoses).<br><br>**Respiratory:** asthma exacerbations; chronic obstructive pulmonary disease (COPD) exacerbations.<br><br>**Cardiovascular:** myocardial infarction; unstable angina; cardiac failure; transient ischaemic attacks; cerebrovascular accidents; venous thromboembolism (pulmonary embolism and deep venous thrombosis).<br><br>**Alcohol:** alcohol-related acute physical and psychological harms.
**E. Objectives, Specific Aims and Rationale**<br>Our overall **aim** is to determine the effects of social distancing and the diversion of healthcare resources to the COVID-19 pandemic on the risk of key adverse acute physical and mental health outcomes in the UK population, and to determine if there

are differences in the burden of these outcomes by: age, sex, ethnicity, socioeconomic deprivation, vulnerable status (i.e. individuals felt to be at particular risk if they become ill with COVID-19), rural or urban location, living alone, and other outcome-specific factors.

Specific **objectives** are to:

1. **Describe changes** in key mental and physical health outcomes (see **Section D**) **before and after lockdown** measures were introduced on 13th March 2020 (with sensitivity analyses looking at alternative time points before 13th March, as the impending events could have already impacted health).
2. Describe if there are **stratum-specific differences** in pre- and post-lockdown burden of key mental and physical health outcomes, after stratifying, where possible, on: age, sex, ethnicity, socioeconomic deprivation, vulnerable status, rural/urban location, partnership, and other outcome-specific factors.
3. Conduct formal statistical tests (generalised linear models and an interrupted time series design) to investigate whether there is **statistical evidence for a difference between pre- and post-lockdown** burden of key mental and physical health outcomes.

We will test six hypotheses:

1. **Hypothesis 1:** Presentations with **diabetic emergencies** (diabetic hyper- and hypoglycaemia, ketoacidosis and diabetic comas) will increase. This increase may be due to reduced routine disease monitoring, reduced access to face-to-face consultations and reduced access to specific therapies, in many cases exacerbated by individuals being categorised as having vulnerable status.
2. **Hypothesis 2:** Consultations for **mental health conditions**, e.g. depression, will reduce during lockdown. The reduction may be due to decreased access to face-to-face consultations, talking therapies, and social distancing and avoidance (reduction in consultations may be accompanied by reduced prescribing). However, consultations for more severe mental health conditions may increase.
3. **Hypothesis 3:** Presentations with **asthma and COPD exacerbations** will increase. These changes may be due to reduced access to face-to-face consultations, regular monitoring, inclusion on the extremely vulnerable list and avoidance behaviour. However, reduced air pollution might reduce exacerbations.
4. **Hypothesis 4:** Presentations with **unstable angina** and **transient ischaemic attacks** will reduce. This will be accompanied by later presentations with **myocardial infarction** and **stroke** leading to worsened outcomes. One of these worsened outcomes will include increased presentations with **heart failure**. One mechanism for these changes is a lack of access to time sensitive interventions.
5. **Hypothesis 5:** Presentations for **venous thromboembolic events including deep venous thrombosis** will initially decrease, accompanied by later increases as a result of reduced physical activity due to lockdown.
6. **Hypothesis 6:** Presentations for **alcohol-related harms** will increase. While for some alcohol consumption may decrease, for others, alcohol consumption may increase and presentations for alcohol related emergencies will increase (initial reports suggest 47% of the UK population now start drinking earlier in the day than they did prior to the lockdown[1]). Reasons for reduced alcohol consumption may include less social drinking and removed access to venues where alcohol is typically consumed (bars, restaurant, pubs), while heightened anxiety, boredom and removal of social constraints (e.g. less concern about social disapproval for hangover, morning drinking) may lead to increased consumption.

However, we expect to see an initial decrease in presentations for all of our outcomes of interest in the early stages of the pandemic due to reduced access to face-to-face consultations, perceived burden on the health service, inclusion on the extremely vulnerable list and avoidance behaviour.

Rationale

The COVID-19 pandemic is likely to indirectly increase physical and psychological health problems. There will inevitably be impacts on non-COVID-19 related healthcare provision as healthcare resources are reallocated to the COVID-19 response and modifications made to methods of care delivery due to social distancing requirements. These changes to healthcare provision may adversely affect physical and psychological health. Psychological health is also likely to be impacted by fears around the COVID-19 pandemic, as well as control measures such as social distancing, closures of social spaces and self-isolation. Lockdown measures will result in reduced access to a wide range of care including face-to-face visits and talking therapies. Understanding these indirect effects will help public health planning over the following months, particularly when/if the COVID-19 pandemic is under control (and if further lockdowns are needed) and could also help inform control measures for future pandemics.

Although there is potentially a huge range of acute diagnoses that could be indirectly linked to the COVID-19 pandemic, we have focused on a number of specific outcomes in this project that could plausibly be affected acutely. We have specifically selected diabetic and cardiovascular emergencies, and exacerbations of respiratory conditions as these individuals are likely to be included on lists of individuals considered extremely vulnerable (and asked to self-isolate to avoid infection),[2] making it difficult for them to access healthcare resources. Psychological health and alcohol use are also likely to be impacted by fears around the COVID-19 pandemic, concerns about employment, as well as control measures (such as mass social distancing, closures of social spaces and self-isolation). Furthermore, existing mental illness may be affected by difficulty accessing medications and talking therapies whilst in self-isolation.

F. Study Background

As of 16th June 2020, the novel Coronavirus disease 2019 (COVID-19) pandemic has been diagnosed in over 8 million individuals with more than 437,000 deaths reported worldwide.[3] Much of the global public health and research focus has understandably focused on prevention of spread of the virus and reducing mortality.

Specific control measures such as mass social distancing, closures of social spaces and self-isolation have been introduced in an effort to control the pandemic. Major planning has aimed at tackling the increased emergency department hospital attendances and admissions to hospital (including high dependency and intensive care units).

However, as healthcare resources are reallocated to the COVID-19 response and modifications made to methods of care delivery due to social distancing requirements, there will inevitably be impacts on non-COVID-19-related healthcare provision, including prevention activities, such as chronic disease monitoring.[4] The reduction in prevention activities, reductions in attendance at general practitioners and emergency departments for non-COVID-19-related health issues and mass social distancing measures may inadvertently worsen the physical and mental health of the population.[5–9] In addition, people may delay seeking care (due to fear of infection, or a perceived need to reduce the burden on healthcare). Mental health is also likely to be affected by fears around the COVID-19 pandemic, employment and financial concerns, as well as control measures such as mass social distancing, closures of social spaces and self-isolation.

Understanding the indirect effects of the pandemic on non-COVID-19 related health outcomes will help public health planning and policy over the following months, particularly when/if the COVID-19 pandemic is under control, or should further lockdowns become necessary.

Therefore, we will investigate key indirect mental and physical health effects of the COVID-19 pandemic to inform resource allocation and drive UK healthcare policy.

G. Study Type

This is an ecological study with **descriptive** and **hypothesis-testing** components.

Our descriptive component will collect, and graphically present, population-level outcomes presented in **Section D** before and during the pandemic in near-real time (updating when new data become available).

Our hypothesis-testing component will be population-level analyses of these data, comparing proportions of events occurring at specific time points after the pandemic to the expected proportion had the pandemic not occurred, based on 3 years' historical trends.

H. Study Design

Our study has a time series design.

The **descriptive component** will graphically display a weekly time series of outcome proportions, from the first week of 2017 to the most current week for which data are available.

We will then formally compare proportions before and after the pandemic in **interrupted time series analyses** to assess changes in burden of key health outcomes and to test our hypotheses.

I. Feasibility counts

We have chosen to use CPRD Aurum data for this study as the Aurum population is larger.[10,11] For all outcomes, we expect to have substantially more than five outcome events each week for each denominator population (**Table 1**). A total of 850 events would equate to an average of 5 events per week over the period considered, January 2017 to May 2020 = approx 170 weeks. The feasibility counts are considerably higher than 850 for all outcomes except for diabetic emergencies in children, where we may need to suppress small event counts or consider aggregating data by months instead of weeks.

Table 1. Feasibility counts for outcomes of interest in CPRD Aurum January 2017 to May 2020.

Outcome of interest	Age group (years)	Number of events (numerator)	Number of people (denominator)
Diabetes			
Diabetic emergencies	<18	631	16,408*
	18+	5,305	825,466*
Mental health			
Anxiety	5-17	123,782	1,563,804
	18+	1,822,827	8,087,715
OCD	5-17	2,186	1,563,804
	18+	76,169	8,087,715
Depression	5-17	78,332	1,563,804
	18+	1,547,307	8,087,715
Anorexia, bulimia, and other feeding disorders	5-17	14,358	1,563,804
	18+	32,346	8,087,715
	5-17	2,198	1,563,804

Schizophrenia, other psychoses, and bipolar disorder	18+	246,625	8,087,715
Self-harm	5-17<br>18+	37,145<br>219,154	1,563,804<br>8,087,715
Respiratory			
Asthma exacerbation	<18<br>18+	31,016<br>173,743	243,736[a]<br>2,339,488[a]
COPD acute exacerbation	40+	152,918	232,833[b]
Cardiovascular			
Myocardial infarction	30+	114,145	6,601,211
Unstable angina	30+	7,926	6,601,211
Cerebrovascular accident	30+	207,348	6,601,211
Transient ischaemic attack	30+	68,333	6,601,211
Heart failure	30+	288,430	6,601,211
Venous thromboembolism	30+	150,116	6,601,211
Alcohol			
Alcohol-related harms	18+	28,497	8,087,715

The number of events recorded since 1st January 2017 were estimated as numerator for different outcomes of interest. The number of people who were alive and registered with GP for at least 1 year in the practice with latest date of data collection on or after 1st Jan 2017 were estimated as denominator except diabetes emergency, asthma acute exacerbation and COPD acute exacerbation.
*Number of people who had a record of diabetes before 1st January 2017.
[a]Number of people who had a record of asthma before 1st January 2017.
[b]Number of people who had a record of COPD before 1st January 2017.

J. Sample size considerations

Our initial analyses will be descriptive only and therefore unaffected by statistical power concerns. However, to preserve confidentiality, we will supress any estimates of weekly proportions of individuals experiencing an outcome where outcome event counts are less than five. We do not expect that we will need to suppress any event counts for estimates of weekly outcomes in the whole study population, since as outlined above (**Section I**) we expect to have more than five outcome events each week for all outcomes under investigation. However, in subsequent analyses, where we stratify results by age, sex, ethnicity, etc (see **Section N**), we may need to suppress some stratum-specific event counts. We may also consider aggregating data by months (instead of weeks) for less common outcomes.

Interrupted time series designs require a sample size per time point, but exact formulae to calculate them do not exist, as they require specification of the total number of time points, the location of the 'interruption' (i.e. when lockdown measures introduced for this study) within the series, the nature of the interruption (for example as a step change or slope change) and the prevalence of the pre-interruption outcome in addition to the anticipated effect size, precision, power and alpha. These extra parameters vary across our planned analyses. Recent work using simulations gives some insight on the effect of these extra parameters, and suggests that in a linear regression model with 48 time points, a late interruption (i.e. beyond the halfway time point), a step change and a pre-interruption prevalence of 3.5%, 3,000 individuals (i.e. denominator population) per time point would have nearly 100% power to detect a two-fold change at the 5% level.[14] Practically, we would extract data from all people experiencing the outcome of interest in a given time period and calculate the proportion they represent of the denominator population (vary depending on outcome, see **Section L**). As detailed in **Section I**, for our study outcomes, sample size will be higher than 3,000 individuals per time point.

K. Planned use of linked data (if applicable):

Demonstrating and quantifying the key acute physical and mental health outcomes that we have chosen to study at population-level is important for public health planning and policy implementation during the pandemic and when/if the COVID-19 pandemic is under control. Evidence for urgent need will immediately help policymakers reallocate healthcare resources after the lockdown is lifted. Using linked data is essential to help us better answer our research questions, we outline specific justifications for each linkage requested below.

Hospital Episode Statistics – admitted patient care (HES APC)
We will only use hospital admissions data in sensitivity analyses where we will restrict to those eligible for HES linkage to more completely capture and accurately date acute outcomes. We will conduct our initial analyses using primary care data only, in order to deliver answers to our research questions rapidly. HES data will be included in sensitivity analyses, once the current lag in HES data is resolved.

If funding permits, we will also explore the use of HES Accident and Emergency data to more fully capture and date outcome events in follow-up sensitivity analyses.

ONS – death data
We will use up to date ONS death data when it becomes available in secondary analyses to capture instances where our outcomes of interest have resulted in death (**Section O**).

Carstairs index

We will use quintiles of practice-level Carstairs as a measure of socioeconomic deprivation (scores are comparable between the different countries of the UK) to explore whether the changes in the burden of outcome measures are different when stratified by deprivation.

Rural-urban classification
We will use the location of the GP practice in a rural or urban area to explore whether the changes in the burden of outcome measures are different in rural and urban areas. In the context of this study, we believe rural-urban practice location and Carstairs will capture distinct aspects.[15–18] It is likely that there are differences in health service provision between rural and urban settings (in terms of geographical access to specialist services) that are not a reflection of socioeconomic deprivation, and there is also evidence suggesting that there is a greater risk of mental illness in urban environments independent of socioeconomic status.[18]

We are aware that the combination of area-level measures we plan to use (Carstairs quintiles and rural-urban classification) may pose a risk of practice re-identification. We therefore plan to use the following **risk mitigation plan**:

1. Two named individuals on the study team (Rohini Mathur and John Tazare) will be nominated to be the only users with access to both area-level measures (Carstairs quintiles and rural-urban classification).
2. The named individuals will process the area-level data and produce aggregate data for use by the rest of the study team.
3. The named individuals have already undertaken user-confidentiality training on risk of re-identification that specifically covers:
    - Confidentiality awareness when dealing with patient-level data (whether anonymised or not);
    - Understanding the conditions detailed in our licence to use CPRD data;
    - What to do if we think that there is a risk of re-identification or other data breach (i.e. contact CPRD immediately for advice).

L. Definition of the Study population
Our overall study population will include individuals with at least one year of registration with a CPRD practice meeting CPRD quality-control standards (i.e. has CPRD acceptable flag) in the study period (January 2017 to latest data collection). Individuals will need to have at least one year of registration to: 1) avoid wrongly excluding individuals from outcome-specific study populations because existing diagnoses have not yet been recorded (i.e. for respiratory and diabetes outcomes); and 2) avoid identifying historical diagnoses (captured in a new-patient consultation) as incident outcomes (i.e. for cardiac failure outcomes).

All individuals will be followed from the latest of: one year from CPRD registration or, for diabetes and respiratory outcomes, from when they meet our definitions for having diabetes or respiratory disease as appropriate (more detail below). Follow-up will end for all study populations at the earliest of the following: no longer registered with GP practice, death, practice stops contributing to CPRD, or the end of the study. We will continue to monitor changes in outcome recording until March 2023 (i.e. 3 years after the initial initiation of lockdown) in order to capture responses to the lifting of lockdown measures, and any subsequent lockdowns.

Study populations (i.e. denominator populations) will vary depending on the outcome being tested:
1. **Diabetic emergencies**: the population will be all individuals (no age limits) with established diagnoses of diabetes mellitus. Individuals will contribute to the study population from the latest of the start of follow-up in the overall population and the date of their first record indicating a diagnosis of diabetes.
2. **Acute mental illness diagnoses:** The study population here will be all children (age 5-17) and adults (≥18) from the overall study population.
3. **Alcohol-related harms:** The study population here will be all adults (≥18) from the overall study population.
4. **Asthma exacerbations**: The study population will be all individuals (age 5+) with a current asthma diagnosis (i.e. asthma code in the last two or three years if <18 years or 18+ years, respectively). Individuals will join the study population from the start of follow-up in the overall population if there is a current asthma diagnosis at this time or from the date of their first record indicating an asthma diagnosis within overall follow-up. Participants will remain in the study until there is no current asthma diagnosis or the end of overall follow-up. They may re-enter the study if there is a later diagnostic code for asthma before the end of overall follow-up. Following an existing definition, individuals 40 years and over with asthma will be considered as likely to have COPD (and therefore not included in the asthma study population [denominator]) if they have a subsequent COPD diagnosis recorded within the two years following the current asthma record.[19,20]
5. **Exacerbations of COPD**: The population will be adults (≥40) with an established diagnosis of COPD and evidence of a smoking history.[21] Individuals will join the study population from the latest of the start of follow-up in the overall population and the date of their first record indicating diagnosis of COPD.
6. **Acute cardiovascular disease emergencies**: The study population here will be all adults (≥30) from the overall study population.

M. Selection of comparison group(s) or controls

This study compares health outcomes before and after the COVID-19 pandemic. Outcomes occurring during the pandemic will be compared to the expected proportions of outcomes had the pandemic not occurred, based on 3-year historical trends.

For acute diabetic and respiratory outcomes, we will calculate the proportion of people with diabetes and respiratory disease (see detail in **Section L**) who experience the outcome of interest each week for the duration of the study period (2017 to latest data available). So, in a given week, for example, we will calculate the proportion of all diabetics who have a record for a diabetic emergency.

For mental illness outcomes, alcohol-related harms and cardiovascular disease outcomes, where the study population will be the Aurum population from 2017 (with age restrictions varying for each outcome), we will calculate the proportion of people in the study population (in outcome-specific age limits, see **Section L**) who experience the outcome of interest.

N. Exposures, Outcomes and Covariates

Exposure

Our exposure will be the introduction of population wide COVID-19 control measures (Friday 13th March 2020). We will also undertake sensitivity analyses going back one month before measures were introduced, and also investigating how disease burden changes as lockdown is lifted or, potentially, in subsequent lockdowns (**Section O**).

Outcomes

We will define all outcomes using morbidity coding initially in primary care only, and then, as up-to-date hospital data becomes available, we will also use hospital record data to more completely capture outcomes in a sensitivity analysis limited to individuals eligible for HES linkage (and to investigate whether any reduction in primary care coding is explained by increases in hospital admissions).

For some outcomes we will define a period during which we will regard further coding for the same outcome as representing the same biological event. We will use different outcome-specific time periods to define outcome events to account for differences in the natural history of the different outcomes under investigation. **Table 2** includes a summary of how we will define our outcome measures.

Table 2. Definition of outcome variables (defined using primary care coding only in our main analyses, and additionally using hospital admissions coding in sensitivity analyses).

Outcome	Definition
Diabetes	
Diabetic emergencies	Records coded with morbidity codes for hyperglycaemia, hypoglycaemia, ketoacidosis, or diabetic coma. If an individual has multiple records for a diabetic emergencies, we will define an acute event based on records separated by a gap of up to **seven days**; if an individual has a subsequent record within the seven days following the first record, the second record will be considered as representing the same event, and so on until there is a gap of more than seven days between subsequent records, at which point the next record will be considered another diabetic emergency event.
Mental health	
Anxiety	Anxiety will be defined by codes for symptoms and diagnoses of: social phobia, agoraphobia, panic disorders, generalized anxiety disorder, and mixed anxiety and depression. We will only count one consultation in a **7-day period** per person (i.e. if an individual has two or more consultations separated by less than 7 days we will only count the first of those consultations, a subsequent consultation recorded 7-days or more from the first record, irrespective of whether there is a intervening record(s) will also be counted). Here we are aiming to capture the number of people consulting each week, and will only count one consultation per person **per week**.
Depression	Depression will be defined using codes for diagnoses of major depressive disorders, dysthymia, mixed anxiety and depression, and adjustment disorders with depressed mood. We will also include codes for depressive symptoms. Our outcome will be the number people consulting each week. As for anxiety, we will only count one consultation per person **per week**.
Self-harm	Self-harm will be defined by codes where the intention to self-harm is explicit (e.g. deliberate self-harm) and include codes of non-suicidal or suicidal self-harm (e.g. attempted suicide). It will also include overdoses with drugs commonly implicated in suicide (e.g. paracetamol).<br>Possible self-harm will be defined as when the intent is unclear (e.g. undetermined, query accidental). As for other mental illness outcomes, we will aim to capture the number of individuals consulting in one week, and only count one consultation per person **per week**.
Serious mental illness	Severe mental illness will be defined by codes for diagnoses of schizophrenia and other psychotic disorders, and bipolar disorders. As for other mental illness outcomes, we will aim to capture the number of individuals consulting in one week, and only count one consultation per person **per week**.
Eating disorders	Eating disorders will be defined as anorexia nervosa, bulimia nervosa, and other specified feeding and eating disorders. As for other mental illness outcomes, we will aim to capture the number of individuals consulting in one week, and only count one consultation per person **per week**.

Obsessive compulsive disorder	Obsessive compulsive disorder will be defined by codes for body dysmorphic disorders, hypochondriasis, hoarding disorder, and body focused repetitive behaviour disorders. As for other mental illness outcomes, we will aim to capture the number of individuals consulting in one week, and only count one consultation per person **per week**. If this outcome is has very low event counts it will be combined with the anxiety outcome.
Respiratory	
Asthma exacerbations	Aasthma exacerbations will be defined as records for morbidity codes for asthma exacerbations and status asthmaticus, or a primary care prescription for an oral corticoseroid.[22] We will define acute events allowing a **14-day** window between successive records (records separated by more than 14 days will be considered to be another event).
COPD exacerbations	Exacerbations of COPD will be defined using morbidity codes in individuals with existing COPD for COPD exacerbations, lower respiratory tract infections, breathlessness or sputum production, or a new prescription for an oral corticosteroid or antibiotic.[23] We will define acute events allowing a **14-day** window between successive records (records separated by more than 14 days will be considered to be another event).
Cardiovascular	
Myocardial infarction	We will define myocardial infarctions using relevant morbidity codes, allowing for a **1-year** window between successive records (records separated by less than one year will be regarded as being part of the same MI event).
Unstable angina	We will define unstable angina using relevant morbidity codes, allowing for a **6-month** window between successive records (records separated by less than six months will be regarded as being part of the same event).
Transient ischaemic attacks	We will define transient ischaemic attacks using relevant morbidity codes, allowing for a **6-month** window between successive records (records separated by less than six months will be regarded as being part of the same event).
Cerebrovascular accident	We will define cerebrovascular accidents using relevant morbidity codes, allowing for a **1-year** window between successive records (records separated by less than one year will be regarding as being part of the same event).
Cardiac failure	Given the complexity with capturing acute events for a chronic condition, we will only count an individual's **first ever diagnosis** with cardiac failure.
Venous thromboembolism (pulmonary embolism and deep venous thrombosis)	We will define venous thromboembolism using relevant morbidity codes, allowing for a **1-year** window between successive records (records separated by less than one year will be regarded as being part of the same event).
Alcohol	
Alcohol-related harms	We will define alcohol-related harms as acute physical and psychological alcohol-related harms, including acute alcoholic pancreatitis, new diagnoses of alcoholic cirrhosis, alcohol-related. We will define acute events allowing a **14-day window** between successive records.

Stratifying variables (covariates)

For all outcomes we will stratify, where possible, on the following variables: age (in 10-year bands), sex, quintile of Carstairs Index of deprivation, rural/urban classification, ethnicity, vulnerable status, geographic region, body mass index (BMI), and relationship status (as a proxy for capturing whether someone lives alone).

We will define 'clinically vulnerable' individuals based on those who would be offered influenza vaccination for medical reasons.[24] Medical reasons for offering influenza vaccination include individuals with: chronic liver disease, chronic kidney disease, malignancy, chronic cardiac disease, chronic respiratory disease, diabetes, chronic neurological disease, transplant recipients, individuals with immunosuppression (e.g. morbidity coding for: human immunodeficiency virus, splenic disorders, sickle cell anaemia, aplastic anaemia, leukaemia, lymphoma, myeloma, bone marrow or stem cell transplants, chemotherapy or radiotherapy; or prescriptions for immunosuppressants). When defining vulnerable status for specific outcomes, we will exclude the outcome under investigation from the vulnerable status definition (e.g. for diabetic emergencies, we will exclude diabetes from the definition of vulnerable status). We will define clinically vulnerable people based on records for any of the medical reasons for influenza vaccination at any time prior to the week of interest. We will vary when records need to be recorded to define vulnerable status in sensitivity analyses (see **Section O**).

We will identify relationship status using primary care coding (we are aware that this may not be a robust measure and will be cautious in interpreting our results).

Where possible we will estimate body mass index using recorded weight and height measures (using the weight measure recorded closest to the week of interest) as we have in previous studies.[25] BMI will be classified using the World Health Organisation categories, i.e., underweight [<18·5 kg/m2], normal weight [18·5–24·9 kg/m2], overweight [25·0–29·9 kg/m2], and obese [≥30·0 kg/m2]). We will also use a missing indicator category if there are no valid records as this will capture something meaningful about consulting behaviour.

We will also stratify by outcome-specific factors outlined in **Table 3.**

Table 3. Outcome-specific stratifying variables

Outcome	Stratifying variables	Definition
Diabetic emergencies	Type I/II diabetes (or type unclassified)	Defined using an algorithm using morbidity coding and insulin prescriptions recorded at any time prior to the week of interest.[26]
	Glycosylated haemoglobin (HbA1C)	Defined as HbA1c <=58/mmol/mol or <58 mmol/mol recorded, using the latest recorded measure recorded between 13 months and 1 month prior to week of interest (to capture baseline blood sugar control, rather than changes related to the acute event). Individuals with no recorded HbA1c within the 13 months to 1 month prior to week of interest will be included in a missing category (people with diabetes should have HbA1c measured at least once a year, so if there is no recent record a missing category will capture something meaningful about consulting behaviour).
Mental illness outcomes	History of common health disorders	Defined using morbidity coding recorded at any time prior to the week of interest.
	History of serious mental illness	
Asthma exacerbations	Asthma severity	Defined using British Thoracic Society (BTS) standards applied to the most recent primary care prescribing records recorded between 13 months and 1 month prior to the week of interest, to capture baseline asthma severity.[27] The BTS stepwise approach (incorporating inhaler class and dose) is a recommended evidence-based method of measuring asthma severity.
	Prescription for a short acting beta-agonist (SABA)	Defined using primary care prescribing records for SABA recorded between 13 months and 1 month prior to the week of interest.
COPD exacerbations	Forced expiratory volume (FEV1)	Defined using spirometry data derived from primary care records, using the latest recorded measure recorded between 19 months and 1 month prior to week of interest (with a missing category included to categorise people with no spirometry records during this period).
Cardiovascular outcomes	History of previous cardiovascular disease.	Defined using morbidity coding recorded at any time prior to the week of interest for: ischaemic heart disease, heart failure (except for analyses where cardiac failure is the outcome), cerebrovascular disease, atrial fibrillation or peripheral vascular disease.
Alcohol-related harms	History of mental illness (common mental disorders or serious mental illness)	Defined using morbidity coding recorded at any time prior to the week of interest.
	Existing chronic alcohol problems	

Please note that for outcomes where an age-restricted subset of the Aurum population is the study population (i.e. for the mental illness, alcohol-related harms and cardiovascular outcomes) we will identify stratifying variables using the CPRD Define tool. We will run a series of Defines to extract files with patient identifiers and event dates for all conditions that are defined using relevant medical or product code lists. This will avoid us extracting the full Aurum population dataset. However, we are aware that currently there is no procedure in place for us to be able to identify BMI using this Define approach, so we may not be able to stratify results by BMI for outcomes where the denominators are the overall study population (i.e. mental illness, alcohol-related harms, and cardiovascular diseaseses). We have discussed this limitation directly with CPRD and we are aware that CPRD are developing a new version of Define that will return a wider range of records, potentially including height and weight measurements. We will use this new Define functionality to identify BMI if it becomes available within the lifespan of the project.

O. Data/ Statistical Analysis

We will collect counts for each outcome from three years prior to the COVID-19 outbreak (2017/2018/2019), as well as all data during and following the pandemic, and calculate proportions of each outcome using the denominator populations (see **Section L** above). We will report the proportion of each outcome aggregated by week, and by week and strata defined by: age, sex, ethnicity, vulnerability status ("vulnerable", "not vulnerable"), relationship status, socioeconomic deprivation, region and urban/rural location. We will plot these proportions against time to describe pre- and post-COVID trends in health outcomes and upload them to LSHTM's website via an interactive data dashboard (supressing any small event counts to preserve confidentiality). We will update the calculations regularly as new data are released (**Appendix 1** includes an example of how we might present our results).

To formally test our hypotheses, we will perform interrupted time series analyses. The interruption will be defined from the initiation of population-wide social distancing measures (13th March 2020). We will produce population-level and stratified estimates of the difference between observed and expected health burden for our selected physical and mental health outcomes during this time. To minimise the risk of false positive findings from multiple statistical analyses, we will report this change at one week, one month and six months post-lockdown (interruption).

To carry out these analyses, we will model the proportion of outcomes within the populations defined in **Sections L and M** each week using a binomial generalised linear model and weight each week's data by the population size. We will use flexible functions of time to control for temporal trends and seasonality. Effect modification by time-invariant or time-varying factors will be evaluated by including interaction terms in the statistical model.

Sensitivity analyses

1. In order to rapidly answer the important research questions asked by our study, our initial analyses will use CPRD data only and not be restricted to those eligible for HES linkage. When up to date HES data becomes available, we will rerun our analyses restricting to those eligible for HES linkage, and **additionally using hospital record data** (from both inpatient admissions, and, if funding permits, accident and emergency records) to more completely capture our outcomes, and also allow us to explore whether any potential decreases in primary care coding are explained by increased hospital admissions.
2. To assess the impact of **including codes for symptoms of anxiety and depression** for anxiety and depression outcome definitions, we will repeat analyses for these outcomes using diagnostic codes only to define outcomes (i.e. excluding symptom codes).
3. We will repeat our analyses allowing alternative **durations between records to define outcome events** (see Table 1) to define outcomes (e.g. in our main analysis we will allow for a 1-year window between successive records to define myocardial infarction events, we will repeat our main analysis changing this to a 6-month window).
4. We will also repeat our formal interrupted time series analyses using **alternative cut points**, that is, rather than focussing on when lockdown measures were introduced, we will instead: i) go back both two weeks and one month before measures were introduced (as health may have already been effected by the impending lockdown); ii) look at graded points as successive measures are lifted (e.g. when guidance was changed to allow individuals regarded as non-essential workers back to work, reopening retail spaces, reopening schools, etc); and iii) when potential subsequent lockdowns are instated.
5. We will repeat our analyses stratified by **vulnerable status** using more complex definitions of vulnerable status. Initially, we will define clinically vulnerable people based on records for any of the medical reasons for influenza vaccination *at any time* prior to the week of interest. In sensitivity analyses – to account for the differing natural history of the different conditions included in the vulnerable definition – we will redefine vulnerable status by *varying the times* when different conditions need to be recorded prior to the week of interest (e.g. individuals will be considered clinically vulnerable if they have a record of being HIV positive at any time prior to the week of interest, but we will only consider individuals as vulnerable if they are prescribed a high-dose oral steroid in the three months preceding the week of interest as the effect of the oral steroid on the immune system will wane over time).

Secondary analyses

1. A limitation of our study is that while more outcomes may occur there may be fewer primary care consultations recorded for them because of reluctance to go to GPs, or to burden the health services. We will explore this limitation by:
    a. Comparing numbers of **consultations (for any condition)** and **number of codes per consultation** between the time periods.
    b. Using the total **number of consultations** in a specific time period as the denominator and examining the proportion of consultations in that period that resulted in a code for each **specific outcome** of interest.
2. As presentations for some conditions are likely to happen later in the illness (due to reduced primary care access as a result of social distancing, fear of infection and perceived burden on health services) we will repeat our analyses using **cause-specific deaths** as an outcome (restricted to those eligible for linkage with ONS data). We will use ONS recording to identify the following specific causes of death: myocardial infarction, stroke (ischaemic or haemorrhagic), diabetic emergencies, asthma, COPD and suicide.
3. To identify the **most severe cases of anxiety and depression**, we will also: 1) ascertain the proportion of individuals who consulted for anxiety who received a selective serotonin receptor inhibitor (SSRI) prescription; and 2) we will quantify the proportion of consultations for depression where an antidepressant was prescribed.

P. Plan for addressing confounding

This study determines population-level change in outcomes after the introduction of population-wide infection control measures, thus we do not expect confounding of this effect to be a major issue. However, temporal trends pre-dating the pandemic will influence these outcomes so our statistical models will finely model seasonality and trends over time. There may also be effect modification by characteristics such as age and socioeconomic deprivation, which we will explore as detailed in **Section O**.

Q. Plans for addressing missing data

We do not anticipate that missing data will be a problem, as we expect that most severe outcomes will be captured in medical records. However, it is likely that some outcomes will not be captured following the onset of the pandemic as individuals may avoid consulting for their symptoms due to concerns about infection or limiting burden on the health service. Therefore, we may see lower rates of some outcomes during the post-lockdown period, but higher rates of the more serious outcomes we are focussing on. A reduction in capture of some of our outcomes may therefore be informative, rather than being regarded as missing data (discussed further in **Section L**).

We plan to use ethnicity as a stratifying variable, it is likely that ethnicity will be missing in some instances.[28] We will therefore include individuals with missing ethnicity as a separate category rather than excluding them from stratified analyses.[29]

R. Patient or user group involvement

Current population health measures inhibit new recruitment and involvement of patients/public and users. Hence, we will liaise with existing groups and relevant charities about involvement with this work. We will also liaise with longstanding patient collaborators.

S. Plans for disseminating and communicating study results, including the presence or absence of any restrictions on the extent and timing of publication

The study findings will be submitted for publication in peer-reviewed scientific journals, and also presented at appropriate conferences and other meetings. We will post findings from our research as news stories on the LSHTM website as they arise. We will also develop a Shiny app (Shiny is an R package for building interactive web apps using R) to our institutional website to more fully share our results (we will supress data for small event counts). We will make our findings available to our infectious disease modelling group, the wider NHS and policy makers. We plan to share our statistical code and simulated data through institutional and personal repositories (e.g., GitHub).

Conflict of interest statement: None known

T. Limitations of the study design, data sources, and analytic methods

There may be under-ascertainment of outcomes, as people are less likely to present to their GP following the pandemic. For example, there could be more anxiety and other outcomes, yet fewer consultations recorded because of reluctance to go to GPs, or to burden the health services. This will need to be considered when interpreting the data. We will explore this limitation by comparing numbers of consultations (for COVID-19 and other conditions) and number of codes per consultation between the time periods (**Section O**).

It will be difficult to assess lower level mental health issues accurately in electronic health records. It will therefore be important to compare our results with those from various population mental health surveys currently being rolled out (e.g. https://www.ucl.ac.uk/news/2020/mar/new-study-psychological-and-social-effects-COVID-19). For mental health outcomes, we will incorporate symptom codes as well as diagnostic codes (as there is known under-use of the specific diagnostic codes in recent years[30,31]). We will also quantify the proportion of mental illness consultations that resulted in prescriptions as a measure of disease severity. We acknowledge that antidepressants have indications other than anxiety and depression (e.g. pain). We will therefore attempt to minimise the potential for misclassification by quantifying the proportion of consultations for anxiety/depression where anxiolytics/antidepressants were prescribed, rather than solely identifying prescriptions. Self-harm is underestimated in primary care records but we do not expect this to vary over time.[32] We will conduct analysis with self-harm ascertained in CPRD Aurum as well as in HES to improve outcome definition.

For respiratory outcomes, acute respiratory illness caused by COVID-19 could lead to asthma and COPD exacerbations, so these may not strictly be indirect effects. Similarly, there have been reports of COVID-related heart disease.[7]

We are aware that by using morbidity coding related to relationship status (as a proxy for isolation), we are unlikely to reliably capture this stratifying variable. We will therefore interpret all results using relationship status with caution.

U. References

1 Winstock A, Davies E, Ferris J, Maier L, Barratt M. Global drug survey: Special edition on COVID-19. 2020 https://www.globaldrugsurvey.com/wp-content/themes/globaldrugsurvey/assets/GDS_COVID-19-GLOBAL_Interim Report-FINAL.pdf.

2 Public Health England. COVID-19: guidance on shielding and protecting people defined on medical grounds as extremely vulnerable. 2020. https://www.gov.uk/government/publications/guidance-on-shielding-and-protecting-extremely-vulnerable-persons-from-covid-19 (accessed March 31, 2020).

3 Johns Hopkins. COVID-19 Map. Johns Hopkins Coronavirus Resour. Cent. 2020. https://coronavirus.jhu.edu/map.html (accessed June 16, 2020).

4 World Health Organization (WHO). COVID-19 significantly impacts health services for noncommunicable diseases. 2020. https://www.who.int/news-room/detail/01-06-2020-covid-19-significantly-impacts-health-services-for-noncommunicable-diseases (accessed June 2, 2020).

5 Yu N, Li W, Kang Q, *et al.* Clinical features and obstetric and neonatal outcomes of pregnant patients with COVID-19 in Wuhan, China: a retrospective, single-centre, descriptive study. *Lancet Infect Dis* 2020. DOI:10.1016/S1473-3099(20)30176-6.

6 Driggin E, Madhavan M V., Bikdeli B, *et al.* Cardiovascular Considerations for Patients, Health Care Workers, and Health Systems During the COVID-19 Pandemic. J. Am. Coll. Cardiol. 2020. DOI:10.1016/j.jacc.2020.03.031.

7 Tam CCF, Cheung KS, Lam S, *et al.* Impact of Coronavirus Disease 2019 (COVID-19) Outbreak on ST-Segment-Elevation Myocardial Infarction Care in Hong Kong, China. Circ. Cardiovasc. Qual. Outcomes. 2020. DOI:10.1161/CIRCOUTCOMES.120.006631.

8 Cluver L, Lachman JM, Sherr L, *et al.* Parenting in a time of COVID-19. Lancet. 2020. DOI:10.1016/S0140-6736(20)30736-4.

9 Douglas M, Katikireddi SV, Taulbut M, McKee M, McCartney G. Mitigating the wider health effects of covid-19 pandemic response. *BMJ* 2020; **369**: 1–6.

10 Wolf A, Dedman D, Campbell J, *et al.* Data resource profile: Clinical Practice Research Datalink (CPRD) Aurum. *Int J Epidemiol* 2019; : 1–8.

11 Herrett E, Gallagher AM, Bhaskaran K, *et al.* Data Resource Profile: Clinical Practice Research Datalink (CPRD). *Int J Epidemiol* 2015; **44**: 1–10.

12 Wing K, Williamson E, Carpenter JR, *et al.* Real-world effects of medications for chronic obstructive pulmonary disease: Protocol for a UK population-based non-interventional cohort study with validation against randomised trial results. *BMJ Open* 2018; **8**: 1–11.

13 Bloom CI, Nissen F, Douglas IJ, Smeeth L, Cullinan P, Quint JK. Exacerbation risk and characterisation of the UK's asthma population from infants to old age. *Thorax* 2018; **73**: 313–20.

14 Hawley S, Ali MS, Berencsi K, Judge A, Prieto-Alhambra D. Sample size and power considerations for ordinary least squares interrupted time series analysis: A simulation study. *Clin Epidemiol* 2019. DOI:10.2147/CLEP.S176723.

15 Weich S, Twigg L, Lewis G. Rural/non-rural differences in rates of common mental disorders in Britain: Prospective multilevel cohort study. *Br J Psychiatry* 2006; **188**: 51–7.

16 Riva M, Curtis S, Gauvin L, Fagg J. Research Centre on Inequalities in Health in Montreal, Canada, and by the Joint International Internship Program from AnEIS Strategic Formation Program and Health Promotion Research team. *Soc Sci Med* 2009; **68**: 654–63.

17 Riva M, Bambra C, Curtis S, Gauvin L. Collective resources or local social inequalities? Examining the social determinants of mental health in rural areas. *Eur J Public Health* 2011; **21**: 197–203.

18 McKenzie K, Murray AL, Booth T. Do urban environments increase the risk of anxiety , depression and psychosis? An epidemiological study. *J Affect Disord* 2013; **150**: 1019–24.

19 Nissen F, Morales DR, Mullerova H, Smeeth L, Douglas IJ, Quint JK. Concomitant diagnosis of asthma and COPD: A quantitative study in UK primary care. *Br J Gen Pract* 2018; **68**: e775–82.

20 Nissen F, Morales DR, Mullerova H, Smeeth L, Douglas IJ, Quint JK. Validation of asthma recording in the Clinical Practice Research Datalink (CPRD). *BMJ Open* 2017; **7**: 1–8.

21 Quint JK, Müllerova H, DiSantostefano RL, *et al.* Validation of chronic obstructive pulmonary disease recording in the Clinical Practice Research Datalink (CPRD-GOLD). *BMJ Open* 2014; **4**: 1–8.

22 Bloom CI, Palmer T, Feary J, Quint JK, Cullinan P. Exacerbation patterns in adults with Asthma in England A population-based study. *Am J Respir Crit Care Med* 2019; **199**: 446–53.

23 Rothnie KJ, Müllerová H, Hurst JR, *et al.* Validation of the recording of acute exacerbations of COPD in UK primary care electronic healthcare records. *PLoS One* 2016; **11**: 1–14.

24 Public Health England. Chapter 19: Influenza. In: The Green Book. 2019: 1–29.

25 Bhaskaran K, Douglas I, Forbes H, Dos-Santos-Silva I, Leon DA, Smeeth L. Body-mass index and risk of 22 specific cancers: a population-based cohort study of 5·24 million UK adults. *Lancet* 2014; **6736**: 60892–8.

26 Eastwood S V., Mathur R, Atkinson M, *et al.* Algorithms for the capture and adjudication of prevalent and incident diabetes in UK Biobank. *PLoS One* 2016; **11**. DOI:10.1371/journal.pone.0162388.

27 Britiish Thoracic Society, SIGN. Sign 158: British guideline on the management of asthma. 2019 https://www.brit-thoracic.org.uk/quality-improvement/guidelines/asthma/.

28 Mathur R, Bhaskaran K, Chaturvedi N, *et al.* Completeness and usability of ethnicity data in UK-based primary care and hospital databases. *J Public Health (Oxf)* 2013; **36**: 684–92.

29 Blake HA, Tomlinson LA, Leyrat C, Carpenter J, Mansfield KE, Williamson EJ. Estimating treatment effects with partially observed covariates using outcome regression with missing indicators. *Biometrical J* 2020; : 1–16.

30 Walters K, Rait G, Griffin M, Buszewicz M, Nazareth I. Recent trends in the incidence of anxiety diagnoses and symptoms in primary care. *PLoS One* 2012; **7**. DOI:10.1371/journal.pone.0041670.

31 Rait G, Walters K, Griffin M, Buszewicz M, Petersen I, Nazareth I. Recent trends in the incidence of recorded depression in primary care. *Br J Psychiatry* 2009; **195**: 520–4.

32 Thomas KH, Davies N, Metcalfe C, Windmeijer F, Martin RM, Gunnell D. Validation of suicide and self-harm records in the clinical practice research datalink. *Br J Clin Pharmacol* 2013; **76**: 145–57.

List of Appendices

Appendix 1: Illustrative example of how we will present our results
Appendix 2: Preliminary code lists (all code lists will be reviewed and finalised using a consensus process)
